# Traditional medical practices for children in five islands from the Society archipelago (French Polynesia)

**DOI:** 10.1186/s13002-023-00617-0

**Published:** 2023-10-18

**Authors:** François Chassagne, Jean-François Butaud, Raimana Ho, Eric Conte, Édouard Hnawia, Phila Raharivelomanana

**Affiliations:** 1 UMR 152 PharmaDev, Université Paul Sabatier, Institut de Recherche pour le Développement (IRD), Toulouse, France; 2 Maison des Sciences de l’Homme du Pacifique (UAR 2503), Université de la Polynésie Française / Centre National de la Recherche Scientifique, Tahiti, French Polynesia; 3 Correspondant du Muséum National d’Histoire Naturelle (PatriNat), Paris & Consultant en foresterie et botanique polynesienne, Tahiti, French Polynesia; 4 UMR 214 EIO, Université de Polynésie Française, IFREMER, ILM, IRD, Faaa, Tahiti French Polynesia; 5 UMR 152 PharmaDev, Institut de Recherche pour le Développement (IRD), Nouméa, New Caledonia

**Keywords:** Pacific, Traditional medicine, Ethnobotany, Safety, Efficacy

## Abstract

**Background:**

Traditional Polynesian medicine for children has been poorly documented, and few data are available on their efficacy and safety. In this context, the aim of this study was to identify traditional practices used for treating children and then assess the efficacy and safety of the most cited remedies by reviewing the literature.

**Methods:**

In 2022, a semi-structured survey was carried out on five islands from the Society archipelago (Bora Bora, Huahine, Moorea, Raiatea, and Tahiti). A total of 86 participants were interviewed including 19 experts in herbalism. A thorough literature review was performed on the most cited plant species to gather the relevant ethnobotanical, pharmacological, and clinical data of each remedy.

**Results:**

Participants mentioned using 469 remedies to treat 69 health disorders. The most represented health categories were digestive system, skin disorders, infectious diseases, and respiratory system. A total of 67 plant species (representing 731 use-reports) were mentioned and *Annona muricata*, *Gardenia taitensis*, and *Hibiscus rosa-sinensis* were the main plants reported. Regarding the safety of cited remedies, one plant (*Microsorum grossum*) showed high risk of toxicity, and its use should be avoided in infants and children.

**Conclusion:**

Our survey confirms the importance of traditional medical practices for children in the Society Islands. A lack of data in children for most cited remedies demonstrate the need for more pharmacological and toxicological research on Polynesian medicinal plants. Finally, the potential risk of toxicity for some cited plant species reported calls for a better information of traditional medicine users and healers.

**Supplementary Information:**

The online version contains supplementary material available at 10.1186/s13002-023-00617-0.

## Introduction

In French Polynesia, traditional medicine is still used today by the population. These practices are still alive because it is part of their heritage and culture just like Polynesians tattoo, dance, and music. It is also important in addressing a lack of access to healthcare in some remote islands. Traditional medicine in Polynesia encompasses rituals, manipulative practices such as massage, bee venom therapy, and the use of remedies mainly based on plants [1–3]. So far, about 200 medicinal plants have been described in French Polynesia, and they are used to treat and prevent a wide range of diseases including digestive, musculoskeletal, nervous, respiratory, skin, and urogenital disorders [4].

In a recent survey, we have noticed that a large part of the traditional medicine from six Polynesian islands were dedicated to infant and children. In this study, childhood illnesses represented the second most cited category of disorders for which traditional medicine was used after musculoskeletal disorders [1]. In another survey, about 65% of tradipractitioners from Raiatea and Tahaa reported that infant and children were their main patients [5].

While traditional Polynesian medicine seems to be mainly used for children, some reports of death and poisonings by herbal remedies and traditional practices were already published. From 1966 to 1972, 183 cases of poisonings (including 56 deaths) due to the use of herbal remedies were recorded in children [4]. More recently, a 16-month-old infant from Bora Bora died of bronchopneumopathy and a superinfected abscess after the victim’s family decided to opt for traditional medicine instead of consulting a medical doctor [6].

As compared to adults, infant and children are more susceptible to the adverse effects and toxicity of traditional practices especially herbal medicines. This vulnerability is due to differences in physiology, immature metabolic enzyme systems, dose per body weight, and a developing central nervous and immune systems [7].

Because the use of herbal remedies for infants and children in French Polynesia is frequent and toxicity events already occurred, we decided to perform a survey aiming to document the traditional practices used to treat children (from 0 to 12 years old). Based on this data, we provided an evaluation of the efficacy and potential toxicity of the most cited practices and herbal remedies.

Our study focuses on five islands from the Society Islands because case reports of poisonings mainly came from these places, and also because these represent the most densely populated islands from French Polynesia.

## Materials and methods

### Study area

The Society Islands are one of the five archipelagos of French Polynesia located in the South Pacific and they cover a land area of about 1600 km^2^. The Society Islands have the largest population from French Polynesia with about 242,726 inhabitants representing 88% of the total population of French Polynesia. Tahitian and French are the two main languages spoken in the area. Administratively, the Society archipelago is subdivided into two groups: the Windward islands (Îles du Vent) and the Leeward islands (Îles Sous-le-Vent). The Windward islands include four islands (i.e., Maiao, Meetia, Moorea, Tahiti) and one atoll (Tetiaroa) which represent 1200 km^2^ of land area in total. The Leeward islands include five islands (i.e., Bora Bora, Huahine, Maupiti, Raiatea, and Tahaa) and four atolls (i.e., Manuae, Mopelia, Motu One, and Tupai) which cover a land area of 408 km^2^ [8].

**Fig. 1:**
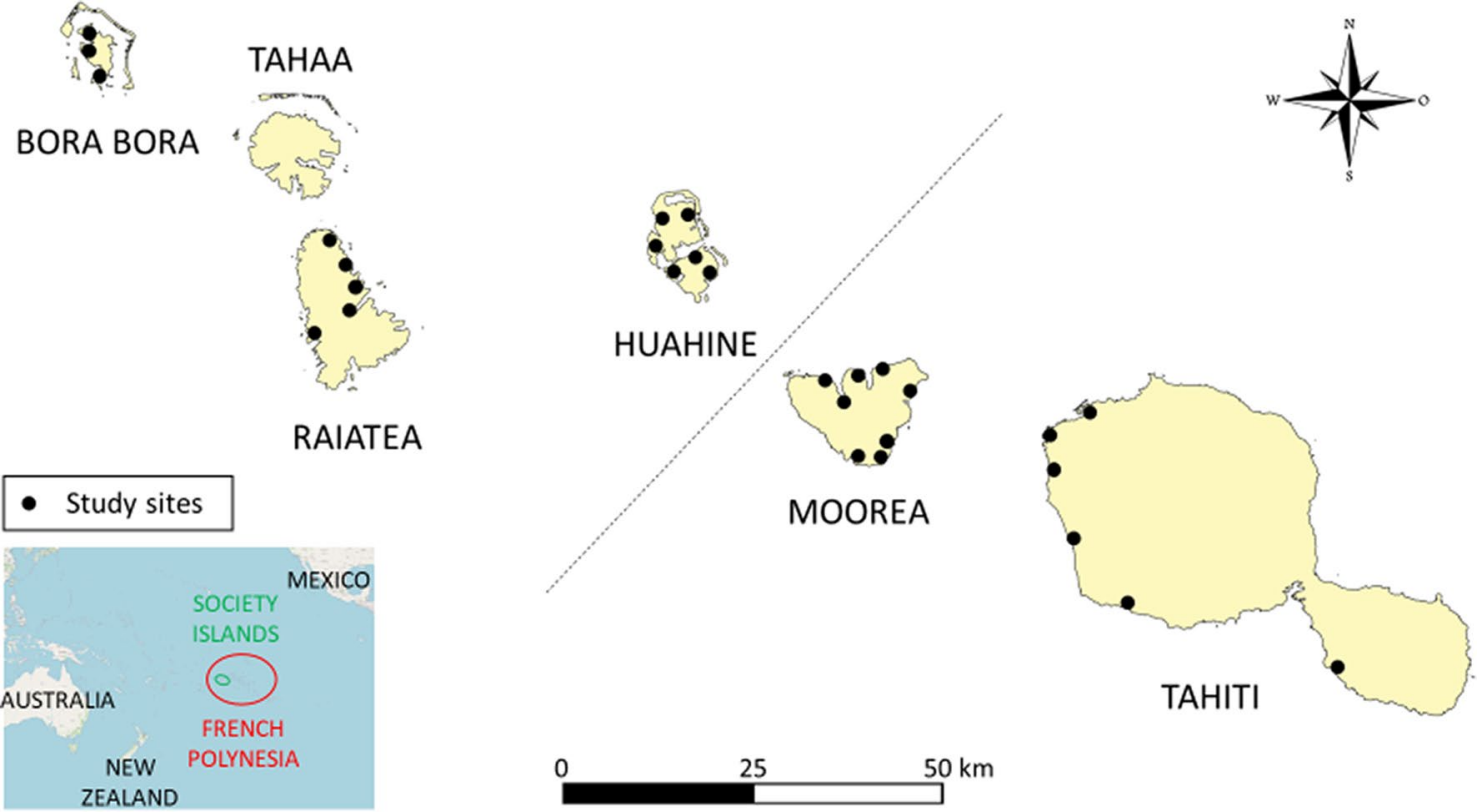
Map showing the locations of the 86 interviews in the five islands (i.e., Bora Bora, Huahine, Moorea, Raiatea, and Tahiti) from the Society archipelago. Fourteen participants were from Bora Bora (i.e. Faanui, Farepiti, Matira, Nunue, Tiipoto, and Vaitape districts), 18 from Huahine (i.e., Fare, Fiti, Haapu, Maeva, Maroe, Tefarerii districts), 17 from Moorea (i.e., Atiha, Haumi, Maharepa, Maatea, Paopao, Papetoai, Pihaena, Teavaro, Temamutu, Urufara, Vaiare districts), 18 from Raiatea (i.e., Avera, Faaroa, Uturoa, Vaiaau districts), and 19 from Tahiti (i.e., Faaa, Paea, Papara, Papeete, Punaauia, Vairao districts)

In this study, a total of five islands was investigated including two in the Windward islands (i.e., Moorea, Tahiti) and three in the Leeward islands (i.e., Bora Bora, Huahine, Raiatea) (Fig. 1). In 2017, Tahiti had the largest population of French Polynesia with about 200,000 inhabitants, while Moorea had 17,700 inhabitants, Bora Bora had 10,600 inhabitants, Huahine had 6,100 inhabitants, and Raiatea had 12,200 inhabitants [9].

### Data collection

This survey was performed in French Polynesia from February to April 2022. Participants were first selected based on the list of tradipractitioners and key informants provided by the Honoea association (non-profit organization aiming to promote and preserve Polynesian medicine), or by using a snow-ball approach. In the latter method, initial participants were randomly selected, and then, they were invited to nominate through their social networks other participants susceptible to contribute to the study. All interviews were conducted in-person, except for one which was conducted online (using WhatsApp application).

Data were collected using a semi-structured questionnaire comprising different parts:

- Socio-demographic data: age, gender, place of residence, place of birth, number of children, occupation, education, religion.
- General knowledge on health disorders treated in children: Tahitian/French names, specificity of affected children (age, gender), symptoms, causes, method of diagnosis, recommendations and avoidance.
- General information on traditional practices: type of treated persons (children only or children and adults, inside or outside family or both, neighbors or patients from other villages/islands), type of used traditional practices (herbal therapies, massage, apitherapy, others), type of treated diseases (all types, some diseases only, others).
- Therapeutic management of each cited health disorder: type of remedies used (herbal therapy, zootherapy including apitherapy, etc.), vernacular name of plant, part of plant, method of preparation, method of administration, posology, local perception of the pharmacology and toxicology of the plant.
- Another question focused on the Polynesian traditional practices known to be dangerous.

### Botanical identification

Plants cited by participants were collected in the field or in homegardens *(Fa’a’apu)* by following a guideline for collection of plant materials previously published [10]. First, photographies of the plant to be collected were taken, and then, information on the plant characteristics, location, and habitat was recorded. Finally, two similar voucher specimens of each plant species were collected and deposited at two different herbariums. One was brought to the Herbier de Polynésie française (PAP), Musée de Tahiti et des îles, Punaauia, Tahiti, Polynésie française, and the other one was deposited at the herbarium from the Jardin Botanique Henri Gaussen (TL), Museum d’Histoire Naturelle, Toulouse, France. Botanical identification of each plant species was realized by the first and second authors of this article. For sterile samples, a botanical identification was performed by comparing them with other samples already present at PAP. The collection numbers of each voucher specimen shown in Table 3 represent the initials of the first author (FC) followed by a unique number of collection. All plant names have been checked and updated according to international and local databases: Plants of the World Online ((https://powo.science.kew.org/), the French inventory of natural heritage (INPN) (https://inpn.mnhn.fr/accueil/index), and the Nadeaud database from French Polynesia (https://nadeaud.ilm.pf/).

### Ethical considerations

A declaration of research regarding access to biological resources (ABS) and associated traditional knowledge was recorded to the Direction of Environment (DIREN) from French Polynesia (n°41/MCE/ENV, January 10th, 2022). Before each interview, an explanation of the survey (objectives, location sites, duration, content of the questionnaires, valorization of research results) was provided, and a prior informed consent was obtained. No personal information (last name, first name, date of birth, personal address) was collected during the survey, which make the responses in the questionnaires not sufficient to identify the participants to the survey. Of note, authors of this article (FC, JFB, RH, PR) are members of a local non-profit organization (Honoea) involved in the promotion and preservation of traditional medicine in French Polynesia.

### Data analysis and visualization

All collected data were compiled in an Excel file. Descriptive analysis was performed based on this Excel database. ICD-11 online browser was used to classify the health disorders cited (https://icd.who.int/browse11/l-m/en).

For Polynesian plant and disease names that participants mentioned, we used the online dictionary of the Tahitian Academy (Fare Vāna'a) to check for correct spellings (http://www.farevanaa.pf/dictionnaire.php).

Use-Reports (UR) were recorded for each plant species and other ingredients following the definition provided by Chellappandian and coauthors [11].

Fidelity Level (FL) was calculated by using the formula described by Friedman and coauthors [12]. FL is an index commonly used to identify plant species dedicated to the treatment of specific disorders. The higher the index, the more the plant is used for a given condition (or a category of disease).

Informant Consensus Factor (ICF) was calculated by following the work from Trotter and Logan [13]. ICF is used to assess consensus among participants for the use of similar plant species in a given disease category. The higher the index, the more convergence was attributed in the type of plants used in a given disease category. ICF ranges from 0 to 1.

The efficacy and toxicity assessment of the most cited remedies was performed based on preclinical studies (in vitro and animal studies) found in the literature. For each most cited remedy, a bibliographic search was realized by searching the electronic database Google Scholar, and by using the following keywords: “scientific name of the plant” AND [“ethnobotany” OR “pharmacology” OR “toxicology” OR “phytochemistry”]. Only scientific articles and reviews published in journals with impact factor above 1 and present in the Scimago database (https://www.scimagojr.com/) were included in the analysis. We organized the ethnobotanical discussion by geographical areas, presenting data on neighboring countries first, then expanding to more distant countries, in order to unveil similar uses that could have been transferred to Polynesians ancestors through their migration from Asia to Polynesia a few thousand years ago.

QGIS software v. 3.30.0 was used to make the map representing the study sites. Layers for French Polynesian islands were found on the website https://www.data.gouv.fr/ under the section “BD Carto PF.” OpenStreetMap was used to make the world map.

## Results and discussion

### Socio-demographic data and classification of healers

In this study, 86 participants were interviewed on the five studied islands. Among them, 36 (41.9%) were from the Windward islands, and 50 (58.1%) were from the Leeward islands (Table 1).

**Table 1:** Socio-demographic characteristics of the 86 participants interviewed in the five islands from the Society archipelago

Characteristics	Frequency	Percent (%)
*Gender*		
Male	8	9.3
Female	78	90.7
*Age*		
20–30 years	5	5.8
31–40 years	11	12.8
41–50 years	13	15.1
51–60 years	29	33.7
61–70 years	20	23.3
71–80 years	8	9.3
*Residence*		
Bora Bora	14	16.3
Huahine	18	20.9
Moorea	17	19.8
Raiatea	18	20.9
Tahiti	19	22.1
*Education*		
Primary School	15	17.4
Secondary School	38	44.2
Vocational diploma	8	9.3
High School	17	19.8
University	5	5.8
ND	3	3.5
*Religion*		
Protestant	49	56.9
Mormon	11	12.8
Catholic	9	10.5
Adventist	9	10.5
Other	7	8.1
ND	1	1.2

*ND* Not documented

The age range of the participants was between 20 and 77 years old with a mean age of 53.9 years old and a median of 56 years old. Most of participants were females (78, 90.7%) and stopped their education before high school (61, 70.9%). The most represented religion was Protestantism with 49 participants (56.9%).

In our study, a high proportion of women were interviewed. Most ethnobotanical studies in French Polynesia have already found this predominance of females over males [1, 2, 5]. Indeed, women (i.e., mothers and grand-mothers) have the role of health careers in the community, especially for children. And they are often in charge of the pharmacopeia dedicated to the care of persons living in their family [14]. Following the Polynesian healers classification previously described [1], 38 (44.2%) participants were categorized as non-specialists, 26 (30.2%) were classified as knowledgeable, and 22 (25.6%) as specialists. Among the specialists, 16 (72.7%) were experts in herbalism only, two (9.1%) were experts in massage only, one (4.5%) was expert in apitherapy only, two (9.1%) were experts in herbalism and massage, and one (4.5%) was expert in herbalism, apitherapy and massage. Out of these 22 specialists, three reported that they were specialized in the treatment of children. Among the knowledgeable persons, eight (30.8%) reported to treat all types of patients (and not just family members), seven (26.9%) were known by other people in the area for being able to heal, four (15.4%) mentioned to treat all types of patients and had a reputation of healing people, four others (15.4%) were in the process of learning, and three (7.7%) had a reputation of healing and possessed a book of homemade remedies.

Mean age of non-specialists was 50.4 years old, 55.5 years old for knowledgeable, and 57.9 years old for specialists. The mean age of specialists was significantly higher than the mean age of non-specialists (p = 0.031). Similar results were obtained in a previous study where specialists were significantly older than the two other groups [1]. This indicates that people with higher knowledge in Polynesian medicine tend to be the eldest.

### Overview of health disorders reported for children

A total of 69 health disorders belonging to 17 different health categories (as defined by the International Classification of Diseases 11^th^) were reported (Table 2). The most represented health category was digestive system (12 cited disorders, 17.6%) followed by skin disorders (10, 14.7%), respiratory system (8, 11.8%), infectious diseases (8, 11.8%), injury, poisoning, and other consequences of external causes (7, 10.3%), and genitourinary system (7, 10.3%). In terms of number of citations by participants, respiratory system ranked first (86 citations, 19.6%), followed by digestive system (76 cit., 17.3%), mental, behavioral or neurodevelopmental disorders (61, 13.9%), skin disorders (46 cit., 10.5%), and injury, poisoning, and other consequences of external causes (43 cit., 9.8%).

**Table 2:** Children illnesses and their disease categories reported during the survey by the 86 participants from the five islands in the Society archipelago

ICD-11 classification	Disorders (English translation)	Disorders (Tahitian and/or French names)	Number and percent of informants citing the disorder
Digestive system			
	Colic	colique (French)	1 (1.2%)
	Diarrhea	hī (Tahitian), diarrhée (French)	3 (3.5%)
	Indigestion	indigestion (French)	1 (1.2%)
	Jaundice	ira re'are'a (Tahitian), jaunisse (French)	2 (2.3%)
	Lip and mouth swelling	vaha pē (Tahitian), bouches et lèvres gonflées (French)	1 (1.2%)
	Lower abdominal disorders	tia (Tahitian)	12 (14%)
	Mouth ulcer	'oromo'o, vaha pē (Tahitian), aphte (French)	4 (4.7%)
	Ranula (salivary cyst)	arero ma'a (Tahitian), double langue (French)	25 (29.1%)
	Rectal prolapse	descente d'organe chez le garçon (French)	1 (1.2%)
	Stomachache	'ōuma pē (Tahitian)	2 (2.3%)
	Teething	niho (Tahitian)	24 (27.9%)
	Yellow lip	lèvres jaunes (French)	1 (1.2%)
Skin disorders			
	Acute blistering skin eruption	cloques partout sur la peau (French)	2 (2.3%)
	Eczema	he'a pa'a (Tahitian), eczema (French)	2 (2.3%)
	Furuncles, abscess, and other disorders with pus exudation	tui (Tahitian), furoncles et abcès (French)	13 (15.1%)
	Heat rash	bourbouille (French)	5 (5.8%)
	Pustular rash	boutons de pus partout sur la peau (French)	1 (1.2%)
	Skin disorders (associated with He'a)	he'a (Tahitian), bobos dû au he'a (French)	12 (14%)
	Skin disorders (not specified)	ma'i 'iri (Tahitian)	7 (8.1%)
	Skin fungal infections	champignons sur la peau (French)	1 (1.2%)
	Skin infections (due to mosquito bite)	infections dues aux piqûres de moustiques (French)	1 (1.2%)
	Skin lesions	tūtu'a (Tahitian)	2 (2.3%)
Respiratory system			
	Asthma	ahopau (Tahitian), asthme (French)	13 (15.1%)
	Bronchitis, productive cough	tūto'o (Tahitian), bronchite, toux grasse (French)	2 (2.3%)
	Cough	hota (Tahitian), toux (French)	23 (26.7%)
	Dyspnea	fati 'ōuma pē (Tahitian), difficulté à respirer (French)	1 (1.2%)
	Nasopharyngitis, cold	hūpē (Tahitian), rhume (French)	3 (3.5%)
	Respiratory disorders	maladies respiratoires (French)	1 (1.2%)
	Sinusitis	nanu (Tahitian), sinusite (French)	35 (40.7%)
	Tonsillitis, sore throat	ma'i 'arapo'a (Tahitian), angine, maux de gorge (French)	8 (9.3%)
Infectious diseases			
	Acute rheumatic fever	rhumatisme articulaire aigu, R.A.A. (French)	1 (1.2%)
	Chickenpox	'ōniho (Tahitian), varicelle (French)	11 (12.8%)
	Filariasis	māriri (Tahitian), filariose (French)	2 (2.3%)
	Gonorrhea	'ōpī, 'ōpītapu (Tahitian), blennoragie (French)	2 (2.3%)
	Infestation by parasitic worms	vers intestinaux (French)	1 (1.2%)
	Measles	rougeole (French)	2 (2.3%)
	Ringworm	seka seka (Tahitian), eczema (French)	3 (3.5%)
	Zoster	māriri 'ōpūpū (Tahitian), zona (French)	1 (1.2%)
Injury, poisoning, and other consequences of external causes			
	Bruise	coups, bleus (French)	1 (1.2%)
	Dislocation	fati rei (Tahitian), déplacement d'os (French)	1 (1.2%)
	Effects of reduced temperature	puta to'eto'e (Tahitian), attraper froid (French)	11 (12.8%)
	Fracture	fati (Tahitian), fracture, enfant mal tenu (French)	18 (20.9%)
	Organ displacement	déplacement d'organes (French)	1 (1.2%)
	Sprain	'o'i, mā'o'i (Tahitian), foulure, entorse (French)	8 (9.3%)
	Wound, cut, burn	blessure, coupure, brûlure, plaies (French)	3 (3.5%)
Genitourinary system			
	Difficulties with micturition	difficulté à uriner (French)	1 (1.2%)
	Menstrual cycle bleeding disorders	he'a (Tahitian), problèmes liés aux régles (French)	3 (3.5%)
	Retractile testis migrans	hua tūpito (Tahitian), testicules remontés (French)	2 (2.3%)
	Scrotal swelling	ira hua (Tahitian), grosseur des testicules (French)	1 (1.2%)
	Urinary incontinence	he'a fa'atahe (Tahitian), incontinence (French)	1 (1.2%)
	Urinary tract infection	he'a (Tahitian), infection urinaire (French)	1 (1.2%)
	Vaginal or urethral discharge	he'a (Tahitian), pertes vaginales ou uréthrales (French)	14 (16.3%)
Musculoskeletal system			
	Muscle soreness	courbature (French)	1 (1.2%)
	Rheumatism	rūmati (Tahitian), rhumatisme (French)	2 (2.3%)
	Scoliosis	scoliose (French)	1 (1.2%)
Developmental anomalies			
	Bones deformity	os malformés (French)	1 (1.2%)
General symptoms			
	Fever	fīva (Tahitian), fièvre (French)	17 (19.8%)
	Headache	māuiui upo'o (Tahitian), maux de tête (French)	3 (3.5%)
Mental, behavioral or neurodevelopmental disorders			
	Hyperactivity	hyperactivité (French)	1 (1.2%)
	Restlessness, irritability, jerk	ira (Tahitian)	60 (69.8%)
Visual system			
	Conjunctivitis	mata vare (Tahitian), conjonctivite (French)	2 (2.3%)
	Eye disorders	ma'i mata (Tahitian), problèmes des yeux (French)	2 (2.3%)
Ear or mastoid process			
	Otitis	tari'a ma'i, tui (Tahitian), otite (French)	13 (15.1%)
Endocrine, nutritional or metabolic diseases			
	Obesity	obésité (French)	2 (2.3%)
Nervous system			
	Epilepsy	hōpi'i, ira hōpi'i (Tahitian), épilepsie (French)	8 (9.3%)
New diseases			
	COVID-19	COVID-19 (French)	4 (4.7%)
Perinatal period			
	Umbilical cord care	pito (Tahitian), soins du nombril (French)	12 (14%)
Substances			
	Detoxifying agent	he'a or pu'a roto (Tahitian), nettoyer les impuretés intérieures (French)	14 (16.3%)

Of the 69 health disorders reported, a group of behavioral symptoms called “ira” and described by participants as infants and children presenting restlessness, irritability, and jerk ranked first in terms of citations (60 participants, 69.8%). Sinusitis ranked second (35, 40.7%), followed by ranula (salivary cyst) (25, 29.1%), teething (24, 27.9%), cough (23, 26.7%), fracture (18, 20.9%), fever (17, 19.8%), and vaginal and urethral discharge (15, 17.4%).

While some disorders could be clearly identified thanks to their Tahitian and/or French names (e.g., cough =“hota”, fever = “fiva”, filariasis = “māriri”, sinusitis = “nanu”), others disorders could not be identified precisely. Indeed, some disorders were named based on the body part affected (e.g., lower abdomen = “tia”, teeth = “niho”, umbilic = “pito”), their causative agents (e.g., to be penetrated by the cold = “puta to'eto'e”), their main symptoms (e.g., discharge or presence of pus = “tui”, yellow body = “ira re’are’a”), and their foreign names (e.g., “rūmati” = rheumatism, “covid” = COVID-19). This specificity of Polynesian diseases classification was already described in others islands such as the Samoan islands and the Society Islands [15, 16].

Another set of disorders called “he’a” was difficult to translate in biomedical terms. “He’a*”* could be categorized into two main health categories: skin disorders and disorders of the genitourinary system (e.g., menstrual bleeding disorders, urinary tract infection, vaginal or urethral discharge). It was defined by participants as something bad/impure inside the body that needs to be expelled. Naturally, these impurities are expelled either by the skin, or by the vaginal or urethral way, and thus, symptoms are skin disorders, leucorrhea, urinary tract infection, or menstrual disorders. The *he’a* category was reported to be transmitted from mother to child. When women give birth and are not previously treated by a *rā’au he’a* (remedy for *he’a*), they transmit diseases through their vagina which induce health disorders on the skin of the babies. Later, the children can also develop vaginal (for females) or urethral (for males) diseases if not treated. To prevent or treat this set of disorders, Polynesian people reported using remedies that clean the body or expel internal impurities, so we classified them as detoxifying agents. The *he’a* category was already described as an important type of diseases in the Society Islands as it is one of the three categories of diseases (along with *ira* and *fati*) being predisposing factors for other diseases. It was defined by French scientists as an “humoral illness” following the medical concepts developed by Hippocrates [16, 17]. The *he’a* category is called “epa” in the Marquesas islands [2]. In Tongan islands, the “kahi” category is reported to be caused by an internal blockage and encompasses various genitourinary disorders similar to those described in the *he’a* category [18].

Regarding the set of disorders called “ira,” it was mainly defined as a group of behavioral disorders including restlessness, irritability, to wake with a start, and convulsion. It was also frequently associated with blue spots on the skin, medically known as Mongolian blue spots. While most of the participants (51, 85%) employed the term “ira” only, a few others added a modifier to precise the type of “ira” treated. For example, “ira 'īriti” was cited by six participants and could be defined as an “ira” with spasms and convulsions. “Ira manu” was cited by five participants and could be defined as an “ira” with children presenting a face or doing movements similar to birds. “Ira re’are’a” was cited by one participant and could be defined as an “ira” with jaundice. “Ira to'eto'e” was cited by one participant and could be defined as an “ira” with cold sweat. Less frequently, the term “ira” was also combined with terms used to define specific disorders such as “nanu” for sinusitis, “niho” for teething and “pito” for umbilic cord care. In these few cases (i.e., *ira nanu*, *ira niho*, *ira tūpito*), no changes in the meaning of the original disorders were noted. The *ira* category is widely known throughout Polynesia (especially, in the Cook, French Polynesian, Tongan, and Samoan islands), and it is also called “ila” in some places (e.g., Samoan and Tongan islands) [15, 19]. In these islands, *ira* (or *ila*) refers to childhood diseases and can also be defined as a mark on the skin such as a mole or a freckle [20]. In the Society Islands, Lemaître reported 20 varieties of *ira*, of which 6 were detailed (i.e., *ira 'īriti*, *ira 'ōfera*, *ira to'eto'e*, *ira vāhi*, *ira vau*, and *ira moe*) [21]. This author defined *ira* as productive of nervous disorders. In the same area, Grépin and Grépin noted that fever, spasms, and nervous symptoms are the main characteristics of *ira* [17]. They also mentioned the blue spots on the skin from the lumbar region as being part of the *ira* category. In the Samoan and Tongan islands, the definition of *ira*/*ila* seems to have slightly different meanings. In Tonga, it was reported that *“ila”* refers to red patches (similar to burns) on the skin of children [19]. In Samoa, it was defined as a childhood diarrhea by some authors [20]. Overall, *ira* represents one of the most important categories of childhood diseases in the area, and thus, Polynesian people have developed a rich pharmacopeia to prevent and treat it.

Further details on the *he’a* and *ira* categories as well as on other ailments are provided in the sections below.

### Overview of Polynesian medical practices for children

Overall, the Polynesian medical practices used for children included biologically based practices (reported by 86 participants, 100%), and manipulative practices (i.e., massage [39 participants, 45.3%], blowing into the mouth [8 participants, 9.3%], pushing on the gingiva [4 participants, 4.7%], and cutting the gingiva [3 participants, 3.5%]). Among the biologically based practices, herbal ingredients rank first in terms of citations (731 UR, 93%), followed by the use of animal ingredients (24 UR, 3.0%), a Polynesian cosmetic product called monoi (23 UR, 2.9%), and other products (i.e., mint alcohol [2 UR], saccharose [2 UR], seawater [2 UR], salt [1 UR.], starch [1 UR], and vinegar [1 UR]). The total number of UR is 787.

A total of 469 remedies were reported to be used for children, of which 347 were unique remedies (meaning that these remedies had different ingredients, different uses, and different part of plants used if the ingredients were similar). Up to 17 ingredients were mixed in the remedies. Of the 347 unique remedies, mono-ingredient remedies were the most represented (145 remedies, 224 UR), followed by two-ingredient remedies (97 remedies, 125 UR), four-ingredient remedies (38 remedies, 44 UR), three-ingredient remedies (36 remedies, 45 UR), and remedies with five and more ingredients (31 remedies, 31 UR). The mean number of remedies cited by participants was 5.4 remedies.

Regarding the number of remedies reported by category of diseases, the set of disorders called “ira” presented the highest number of cited remedies (50 remedies, 95 UR), followed by sinusitis (30 remedies, 39 UR), ranula (salivary cyst) (17 remedies, 27 UR), cough (15 remedies, 25 UR), fracture (14 remedies, 21 UR), otitis (13 remedies, 15 UR), and lower abdominal disorders (13 remedies, 13 UR).

#### Herbal ingredients

A total of 67 plant species (representing 731 UR) belonging to 40 botanical families were identified (Table 3). The ten most frequently cited plant species were *Cocos nucifera* (47 participants citing the plants, 57 UR [including different citations by the same participant]), *Gardenia taitensis* (40 participants, 56 UR), *Annona muricata* (33 participants, 35 UR), *Hibiscus rosa-sinensis* “Carnation” (33 participants, 36 UR), *Saccharum officinarum* (33 participants, 52 UR), *Citrus x aurantiifolia* (30 participants, 40 UR), *Curcuma longa* (29 participants, 35 UR), *Cordyline fruticosa* (25 participants, 27 UR), *Microsorum grossum* (24 participants, 27 UR), and *Spondias dulcis* (23 participants, 28 UR). Among these plants, two *(Citrus x aurantiifolia*, *Saccharum officinarum*) can be considered as excipients as they were mainly used in combinations with other plants. Indeed, remedies including *Citrus x aurantiifolia* or *Saccharum officinarum* were multi-ingredients remedies in 97.5 and 96% of the cases, respectively. *Citrus x aurantiifolia*, which is a modern introduction and so a newly incorporated ingredients in Polynesian TM, may be considered as vitamin C provider and flavor enhancer, while *Saccharum officinarum* can be considered as a sweetener. Although *Cocos nucifera* was also employed as an excipient in most of the remedies, nine out of 43 remedies (17.3%) consisted of *Cocos nucifera* only, suggesting the role of this plant as a bioactive ingredient. Pétard already reported that coconut oil, coconut water, and sugarcane juice are mainly used as excipients [4]. This author also mentioned coconut oil as a bioactive ingredient, especially for its purgative property. Regarding lime juice, the same author reported similar findings and confirmed it is mainly used in combination with other plants. However, its role as an excipient is not clearly mentioned.

**Table 3:** Ethnobotanical data of the 67 plant species recorded from the 86 participants in the five islands from the Society archipelago

Scientific name	Botanical family	Voucher No	Origin	Tahitian name	Number of persons citing the plant	Type of disorders treated	Parts used	Method of preparation	Method of administration	Number of UR for each disorder treated
*Achyranthes aspera* L. var. *aspera*	Amaranthaceae	FC560	Pol	’āerofai	3	Detoxifying agent (he'a)	Leafbud	Crush (in mixture), fermentation	Oral	1
						Ranula (salivary cyst)	Leafbud	Crush	Oral	1
						Sprain	Leaf	Crush	Local application	1
*Adenostemma viscosum* J.R.Forst. & G.Forst	Asteraceae	FC557	Ind	vaianu	6	Detoxifying agent (he'a)	Leafbud	Crush (in mixture), fermentation	Oral	2
						Diarrhea	Leafbud	Crush (in mixture)	Oral	1
						Dislocation	Stem	Crush (in mixture)	Local application	1
						Fracture	Leaf	Crush (in mixture)	ND	1
						Sinusitis	Stem	Crush (in mixture)	Oral	1
*Aleurites moluccanus* (L.) Willd	Euphorbiaceae	FC535	Pol	ti'a'iri	7	Lower abdominal disorders	Flower/Seed	Crush (in mixture)	Oral/Massage	2
						Skin disorders	Bark/Seed	Crush	Bath/Local application	2
						Detoxifying agent (he'a)	Seed	Crush (in mixture), fermentation	Oral	1
						Mouth ulcer	Bark	Crush	Local application	1
						Otitis	Flower	Crush (in mixture)	Oral	1
*Allium cepa* L	Amaryllidaceae	NC	Mod	oignon (French)	1	Asthma	Bulb	Crush (in mixture)	Oral	1
*Allium sativum* L	Amaryllidaceae	NC	Mod	ail (French)	1	Otitis	Bulb	Crush (in mixture)	Local application	1
*Aloe vera* (L.) Burm.f	Asphodelaceae	FC540	Mod	aloé (French)	4	COVID-19	Whole leaf	Crush (in mixture)	Oral	2
						Bruise	Leaf (inner gel)	None	Local application	1
						Eye disorders	Leaf	Crush (in mixture)	Local application	1
*Alpinia purpurata* (Vieill.) K.Schum	Zingiberaceae	FC547	Mod	'ōpuhi	2	Restlessness, irritability, jerk	Leaf	Boil	Bath	2
*Annona muricata* L	Annonaceae	FC490	Mod	corossol (French), tōtara	33	Restlessness, irritability, jerk	Leaf	Boil (alone or in mixture)	Bath/Bath, local application/Bath, local application, massage/Bath, local application, massage, water spraying/Bath, massage/Bath, massage, oral/Bath, massage, water spraying/Bath, oral/Local application, massage, oral/Massage, water spraying	33
						Chicken pox	Leaf	Boil	Bath/Oral, local application, massage	2
						Epilepsy	Leaf	Boil	Bath, local application	1
						Fracture	Leaf	Boil (in mixture)	Bath	1
						Furuncles, abscess and others disorders with pus exudation	Leaf	Boil (in mixture)	Massage, water spraying	1
						Nasopharyngitis, cold	Leaf	Boil (in mixture)	Bath, steam bath	1
						Skin disorders	Leaf	Boil	Bath	1
*Artocarpus altilis* (Parkinson) Fosberg	Moraceae	FC486	Pol	’uru, maiore	4	Sprain	Sap	None (alone or in mixture)	Local application	2
						Stomachache	Leafbud	Crush (in mixture)	Oral	2
*Artocarpus altilis* (Parkinson) Fosberg	Moraceae	FC551	Pol	’uru pae'a	8	Fracture	Sap	None (in mixture)	Local application	2
						Lower abdominal disorders	Leafbud	Crush (in mixture)	Oral	2
						Asthma	Leafstalk	Crush	Oral	1
						Dyspnea	Leafstalk	Crush (in mixture)	Oral	1
						Sinusitis	Leafbud	Crush (in mixture)	Oral, massage	1
						Umbilical cord care	Leafbud	Crush	Oral	1
*Barringtonia asiatica* (L.) Kurz	Lecythidaceae	FC503	Ind	hotu, hutu	2	Fracture	Seed	Crush (in mixture)	Local application	1
						Zoster	Seed	Crush (in mixture)	Local application	1
*Calophyllum inophyllum* L	Calophyllaceae	FC538	Pol	tāmanu,’ati	12	Chicken pox	Leaf	Boil/Crush	Bath	7
						Measles	Leaf	Crush	Bath	2
						Detoxifying agent (he'a)	Bark	Crush (in mixture)	Oral	1
						Furuncles, abscess and others disorders with pus exudation	Leaf	Crush	Bath	1
						Pustular rash	Leaf	Boil (in mixture)	Bath	1
						Skin disorders	Leaf	Crush	Bath	1
						Skin fungal infections	Leaf	Boil	Bath	1
*Capsicum frutescens* L	Solanaceae	NC	Mod	’ōporo	4	COVID-19	Fruit	Crush (in mixture)	Oral	2
						Detoxifying agent (he'a)	Fruit	Crush (in mixture)	Oral	1
						Detoxifying agent (puaroto)	Fruit	Crush (in mixture)	Oral	1
						Effects of reduced temperature	Fruit	Crush (in mixture)	Oral	1
						Restlessness, irritability, jerk	Fruit	Crush (in mixture)	Oral	1
*Carica papaya* L	Caricaceae	FC492	Mod	’ī'ītā	1	Fracture	Fruit (unripe)	Grate	Local application	1
*Casuarina equisetifolia* L	Casuarinaceae	FC539	Ind	’aito	2	Acute rheumatic fever	Leaf	Boil	Oral	1
						Difficulties with micturition	Leaf	Boil	Oral	1
*Catharanthus roseus* (L.) G.Don	Apocynaceae	FC518	Mod	pervenche (French)	1	Infestation by parasitic worms	Flower	Boil	Oral	1
*Centotheca lappacea* (L.) Desv	Poaceae	FC565	Ind	’ohe'ohe, piripiri mou’a,’ofe'ofe,	1	Asthma	Leafbud	Crush (in mixture)	Oral	1
*Citrus* × *sinensis* (L.) Osbeck	Rutaceae	NC	Mod	’ānani	2	Teething	Leaf	Boil (in mixture)	Bath, massage	1
						Sinusitis	Fruit	Press	Oral	1
*Citrus x aurantiifolia* (Christm.) Swingle	Rutaceae	FC495	Mod	tāporo	30	Detoxifying agent (he'a)	Fruit	Press (in mixture)	Oral	7
						Vaginal and urethral discharge	Fruit	Press (in mixture)	Oral	6
						Cough	Fruit	Press (in mixture)	Oral	3
						Lower abdominal disorders	Fruit	Press (in mixture)	Oral	3
						Menstrual cycle bleeding disorders	Fruit	Press (in mixture)	Oral	3
						Skin disorders (he'a)	Fruit	Press (in mixture)	Oral	3
						COVID-19	Fruit	Press (in mixture)	Oral	2
						Eczema	Fruit	Press (in mixture)	Oral	2
						Fracture	Fruit	Press (in mixture)	Local application	2
						Diarrhea	Leafbud	Press (in mixture)	Oral	1
						Fever	Fruit	Boil (in mixture)	Oral	1
						Flu	Fruit	Press (in mixture)	Oral	1
						Ranula (salivary cyst)	Fruit	Press (in mixture)	Oral	1
						Restlessness, irritability, jerk	Fruit	Boil (in mixture)	Oral, Bath	1
						Urinary incontinence	Fruit	Press (in mixture)	Oral	1
*Cocos nucifera* L	Arecaceae	FC487	Pol	ha'ari	47	Sinusitis	Coconut milk/Coconut water	Cook (in mixture)/Crush (in mixture)/Boil	Oral/Oral, massage/Local application	11
						Asthma	Bark/Coconut sprout/Coconut water	Cook (in mixture)/Crush (in mixture)	Oral	6
						Fever	Coconut water	Crush (in mixture)/Fermentation/None	Oral/Oral, local application	6
						Detoxifying agent (he'a)	Coconut water	Crush (in mixture)/Crush (in mixture), fermentation	Oral	5
						Lower abdominal disorders	Coconut water	Crush (in mixture)/None	Massage/Oral	4
						Skin disorders (other)	Coconut milk/Coconut water	Cook (in mixture)	Local application/Oral	3
						Fracture	Coconut water	Crush (in mixture)/Fermentation	Oral	2
						Furuncles, abscess and others disorders with pus exudation	Coconut milk	Cook (in mixture)	Oral/Local application	2
						Otitis	Coconut milk/Coconut water	Cook (in mixture)/Crush (in mixture)	Oral	2
						Skin disorders (he'a)	Coconut milk/Fruit	Boil/Crush (in mixture)	Oral	2
						Conjunctivitis	Coconut water	Crush (in mixture)	Local application	1
						Cough	Coconut water	Crush (in mixture)	Oral	1
						Cryptorchidism	Coconut water	Crush (in mixture)	Oral	1
						Dislocation	Coconut water	Crush (in mixture)	Local application	1
						Effects of reduced temperature	Leaf	Boil (in mixture)	Steam bath	1
						Heat rash	Coconut milk	None	Bath	1
						Ranula (salivary cyst)	Coconut water	Crush (in mixture)	Oral	1
						Sprain	Coconut water	Crush (in mixture)	Local application	1
						Stomachache	Coconut water	Crush (in mixture)	Oral	1
						Umbilical cord care	Coconut sprout	Crush (in mixture)	Oral	1
						Vaginal and urethral discharge	Coconut water	Crush (in mixture)	Oral	1
						Zoster	Coconut milk	Crush (in mixture)	Local application	1
*Codiaeum variegatum* (L.) A.Juss	Euphorbiaceae	FC561	Mod	croton	4	Fracture	Leaf	Boil	Bath	4
*Coffea arabica* L	Rubiaceae	NC	Mod	taofe, caféier (French)	2	Effects of reduced temperature	Leaf	Boil	Bath/Steam bath	2
*Coleus amboinicus* Lour	Lamiaceae	FC562	Mod	niauri	4	Cough	Leaf	Boil	Bath, massage	1
						Nasopharyngitis, cold	Leaf	Boil	Bath, steam bath	1
						Otitis	Leaf	Crush (in mixture)	Smell	1
						Restlessness, irritability, jerk	Leaf	Boil	Bath, steam bath	1
						Sinusitis	Leaf	Crush (in mixture)	Smell	1
						Wound, cut, burn	Leaf	Crush	Local application	1
*Coleus barbatus* (Andrews) Benth. ex G.Don	Lamiaceae	FC516	Mod	plante doliprane/sauge doliprane	1	Fever	Leaf	Crush (in mixture)	Oral	1
*Coleus scutellarioides* (L.) Benth	Lamiaceae	FC534	Mod	terevete	8	Fracture	Leaf	Boil/Crush (in mixture)/Heat (in mixture)	Bath/Local application	5
						Sprain	Leaf	Crush (in mixture)	Local application	3
						Furuncles, abscess and others disorders with pus exudation	Leaf	Crush (in mixture)	Local application	1
*Colocasia esculenta* (L.) Schott	Araceae	FC524	Pol	taro	1	Fracture	Leafbud	Heat (in mixture)	Local application	1
*Cordia subcordata* Lam	Boraginaceae	FC504	Ind	tou	21	Skin disorders (he'a)	Leaf	Boil/Crush (in mixture)	Bath/Oral/Oral, local application	5
						Asthma	Leaf	Crush (in mixture)	Oral	3
						Cough	Leaf	Crush (in mixture)	Oral	2
						Bronchitis, productive cough	Leaf	Crush (in mixture)	Oral	1
						Conjunctivitis	Leaf	Crush	Local application	1
						Detoxifying agent (he'a)	Bark, leaf	Crush (in mixture), fermentation	Oral	1
						Fracture	Leaf	Crush (in mixture)	Oral	1
						Furuncles, abscess and others disorders with pus exudation	Bark	Crush (in mixture)	Oral	1
						Heat rash	Leaf	Crush	Bath	1
						Indigestion	Leaf	Crush	Oral	1
						Menstrual cycle bleeding disorders	Leaf	Crush (in mixture)	Oral	1
						Restlessness, irritability, jerk	Leaf	Crush (in mixture)	Oral	1
						Sinusitis	Leaf	Crush (in mixture)	Oral	1
						Skin lesions	Leaf	Crush	Bath, Oral	1
						Vaginal and urethral discharge	Leaf	Crush (in mixture)	Oral	1
*Cordyline fruticosa* (L.) A.Chev	Asparagaceae	FC484	Pol	’autī	25	Restlessness, irritability, jerk	Leaf	Boil/Boil (in mixture)/Rub in hot water	Bath, local application, massage, water spraying/Bath, massage/Bath/Oral, bath/Oral, massage, water spraying	6
						Teething	Leaf	Boil/Crush, heat/Rub	Bath/Bath, local application/Bath, massage	4
						Lower abdominal disorders	Leaf/Leafbud	Boil/Crush (in mixture)	Oral	3
						Sinusitis	Leaf/Leafbud	Crush (in mixture)	Oral/Oral, massage	3
						Colic	Leafbud	None	Local application	1
						Detoxifying agent (he'a)	Leafbud	Crush (in mixture)	Oral	1
						Epilepsy	Leafbud	Crush (in mixture)	Oral	1
						Fever	Leaf	Rub in water	Bath	1
						Fever, muscle soreness	Leaf	None	Local application	1
						Otitis	Leaf	ND	Oral	1
						Sprain	Leafbud	Crush (in mixture)	Local application	1
						Ranula (salivary cyst)	Leafbud	Crush (in mixture)	Oral	1
						Umbilical cord care	Leafbud	Crush, press	Local application	1
						Zoster	Leaf	Crush (in mixture)	Local application	1
*Curcuma longa* L	Zingiberaceae	FC522	Pol	re'a tahiti	29	Detoxifying agent (he'a)	Rhizome	Crush/Crush (in mixture)/Crush (in mixture), fermentation	Oral/Local application	9
						Vaginal and urethral discharge	Rhizome	Crush (in mixture)	Oral	8
						COVID-19	Rhizome	Crush (in mixture)	Oral	2
						Eczema	Rhizome	Crush (in mixture)	Oral	2
						Skin disorders (he'a)	Rhizome	Crush (in mixture)	Oral	2
						Cough	Rhizome	Crush (in mixture)	Oral	1
						Detoxifying agent (pu'a roto)	Rhizome	Crush (in mixture)	Oral	1
						Effects of reduced temperature	Rhizome	Crush (in mixture)	Oral	1
						Flu	Rhizome	Crush (in mixture)	Oral	1
						Heat rash	Rhizome	Crush	Oral	1
						Lower abdominal disorders	Rhizome	Crush (in mixture)	Oral	1
						Menstrual cycle bleeding disorders	Rhizome	Crush (in mixture)	Oral	1
						Restlessness, irritability, jerk	Rhizome	Crush (in mixture)	Oral	1
						Stomachache	Rhizome	Crush (in mixture)	Oral	1
						Urinary incontinence	Rhizome	Crush (in mixture)	Oral	1
						Urinary tract infection	Rhizome	Crush (in mixture)	Oral	1
						Vaginal prurit	Rhizome	Crush (in mixture)	Oral	1
						Wound, cut, burn	Rhizome	Crush	Local application	1
*Cyperus javanicus* Houtt	Cyperaceae	FC537	Ind	mō’u	6	Restlessness, irritability, jerk	Inflorescence stem	Boil/Boil (in mixture)	Bath/Oral, bath	4
						Fracture	ND	Boil (in mixture)	Oral	1
						Sinusitis	Inflorescence	Heat (in mixture)	Oral, massage	1
*Cyperus mindorensis* (Steud.) Huygh	Cyperaceae	FC566	Pol	mō’u upo’o	1	Asthma	Whole plant	Crush (in mixture)	Oral	1
*Davallia solida* (G.Forst.) Sw. var. *solida*	Davalliaceae	FC555	Ind	ti'ati'amou'a, titi	10	Fracture	Rhizome	Boil (in mixture)/Crush (in mixture)	Bath/Local application/Oral	4
						Asthma	Rhizome	Crush (in mixture)	Oral	2
						Stomachache	Rhizome	Crush (in mixture)	Oral	2
						Cough	Rhizome	Crush (in mixture)	Oral	1
						Effects of reduced temperature	Leaf	Boil (in mixture)	Steam bath	1
						Sinusitis	Rhizome	Press	Oral	1
						Teething	Rhizome	Crush (in mixture)	Oral	1
*Dichrocephala integrifolia* (L.f.) Kuntze	Asteraceae	NC	Pol	ta'ata'ahiara	5	Cryptorchidism	Whole plant	Crush (in mixture)	Oral	1
						Detoxifying agent (he'a)	Whole plant	Crush (in mixture), fermentation	Oral	1
						Dislocation	Whole plant	Crush (in mixture)	Local application	1
						Lower abdominal disorders	Leafbud	Crush (in mixture)	Oral	1
						Umbilical cord care	Leafbud	Crush (in mixture)	Oral	1
*Euphorbia hirta* L	Euphorbiaceae	FC502	Mod	‘ea’ea	1	Bronchitis, productive cough	Leaf	Chew	Oral	1
*Ficus prolixa* G.Forst	Moraceae	FC563	Ind	’ōrā	3	Pustular rash	Leaf	Boil (in mixture)	Bath	1
						Skin disorders (he'a)	Leaf	Boil	Bath	1
						Sinusitis	ND	Crush (in mixture)	ND	1
*Ficus tinctoria* G.Forst	Moraceae	FC554	Pol	mati	7	Skin disorders (he'a)	Fig (sap)	Crush (in mixture)	Oral/Oral, local application	3
						Eczema	Fig	Boil	Bath	1
						Furuncles, abscess and others disorders with pus exudation	Fig (sap)	Crush (in mixture)	Oral	1
						Menstrual cycle bleeding disorders	Fig (sap)	Crush (in mixture)	Oral	1
						Restlessness, irritability, jerk	Fig (sap)	Crush (in mixture)	Oral	1
*Gardenia taitensis* DC	Rubiaceae	FC493	Pol	tiare tahiti	40	Restlessness, irritability, jerk	Leaf/Flowerbud	Boil (in mixture)/Crush (in mixture)	Bath/Bath, local application, massage/Bath, massage/Bath, massage, oral/Bath, massage, oral, water, spraying/Bath, oral/Local application/Massage, water spraying/Oral/Oral, local application, massage/Smell	28
						Asthma	Flower/Flowerbud	Boil (in mixture)/Crush (in mixture)	Oral	3
						Cough	Flowerbud/Leaf	Boil (in mixture)/Crush (in mixture)	Oral	3
						Fracture	Flowerbud/Leaf	Boil (in mixture)/Crush (in mixture)	Bath/Oral	3
						Umbilical cord care	Flowerbud/Leaf	Crush (in mixture)	Oral/Oral, bath, water spraying/Oral, local application	3
						Filariasis	Flowerbud	Boil/Crush (in mixture)	Local application/Oral, local application	2
						Lower abdominal disorders	Flowerbud	Crush (in mixture)	Bath	2
						Sinusitis	Leaf	Boil (in mixture)/Crush	Bath, massage/Smell	2
						Skin disorders (he'a)	Flowerbud/Leaf	Crush (in mixture)	Oral	2
						Teething	Leaf	Boil (in mixture)	Bath/Oral, local application	2
						Bronchitis, productive cough	Leaf	Crush (in mixture)	Oral	1
						Dyspnea	Flowerbud	Crush (in mixture)	Oral	1
						Eczema	Flowerbud	Crush (in mixture)	Oral	1
						Furuncles, abscess and others disorders with pus exudation	Leaf	Crush (in mixture)	Massage, water spraying	1
						Otitis	Flower	Crush (in mixture)	Massage, water spraying	1
						Scrotal swelling	Flowerbud	Crush (in mixture)	Oral	1
						Stomachache	Flowerbud	Crush (in mixture)	Oral	1
*Heliotropium arboreum* (Blanco) Mabb	Boraginaceae	FC521	Ind	tāhinu	14	Teething	Leaf/Leafbud	Crush	Local application/Oral	6
						Ranula (salivary cyst)	Leafbud	Crush	Oral	5
						Cough	Leaf/Leafbud	Crush	Gargling/Oral	3
						Tonsillitis, sore throat	Leaf/Leafbud	Crush	Gargling/Oral	2
						Sinusitis	Leafbud	Crush	Oral	1
						Skin disorders (he'a)	Leafbud	Crush (in mixture)	Oral	1
						Wound, cut, burn	Leafbud	Crush	Local application	1
*Hibiscus x archeri* W.Watson	Malvaceae	FC514	Mod	’aute	2	Restlessness, irritability, jerk	Leaf	Boil	Bath, massage/Bath, massage, oral	2
*Hibiscus rosa-sinensis* L. “Carnation”	Malvaceae	FC531	Mod	'aute 'ū'umu	33	Restlessness, irritability, jerk	Flower/Leaf	Boil/Boil (in mixture)	Bath/Bath, local application/Bath, massage/Bath, massage, oral/Bath, massage, oral, water spraying/Bath, oral/Local application, massage, oral/Massage, water spraying/Steam bath	29
						Teething	Leaf	Boil (in mixture)	Bath/Bath, oral, water spraying	2
						Fever	Flower	Boil (in mixture)	Oral	1
						Furuncles, abscess and others disorders with pus exudation	Leaf	Boil (in mixture)	Massage, water spraying	1
						Menstrual cycle bleeding disorders	Flower	Boil (in mixture)	Oral	1
						Nasopharyngitis, cold	Leaf	Boil	Steam bath	1
						Scrotal swelling	Leaf	Boil (in mixture)	Bath	1
*Hibiscus tiliaceus* L	Malvaceae	FC509	Ind	pūrau	7	Chicken pox	Flower	Boil	Bath	2
						Detoxifying agent (he'a)	Bark, leafbud	Crush, fermentation	Oral	1
						Sinusitis	Bark	Cook (in mixture)	Oral	1
						Skin disorders (not specified)	Flower	Boil	Bath	1
						Skin infections (due to mosquito bites)	Flower	Boil	Bath	1
						Sprain	Root	Crush (in mixture)	Local application	1
*Inocarpus fagifer* (Parkinson) Fosberg	Fabaceae	FC523	Pol	māpē	2	Restlessness, irritability, jerk	Leaf	Boil (in mixture)	Bath, massage	2
*Leucas decemdentata* (Willd.) Sm	Lamiaceae	FC549	Pol	niu	8	Sinusitis	Aerial part/Stem/Whole plant	Crush (in mixture)/Crush and heat (in mixture)	Oral/Oral, massage	3
						Cryptorchidism	Whole plant	Crush (in mixture)	Oral	1
						Detoxifying agent (he'a)	Whole plant	Crush, fermentation (in mixture)	Oral	1
						Dislocation	Whole plant	Crush (in mixture)	Local application	1
						Fracture	Leaf	Boil (in mixture)	ND	1
						Furuncles, abscess and others disorders with pus exudation	Aerial part	Crush (in mixture)	Oral	1
*Macropiper latifolium* (L.f.) Miq	Piperaceae	FC559	Ind	’ava'ava raro	1	Epilepsy	Leaf	Boil	Bath	1
*Mangifera indica* L	Anacardiaceae	FC500	Mod	vī popa'ā 'ōhure pi'o	1	Fever	Fruit (unripe)	Grate	Local application, oral	1
*Mentha* sp.	Lamiaceae	NC	Mod	’ōtime	4	Restlessness, irritability, jerk	Leaf/Whole plant	Boil/Boil (in mixture)	Bath/Bath, oral	2
						Sinusitis	Aerial part/Leaf	Crush/Crush (in mixture)	Oral	2
*Microsorum grossum* (Langsd. & Fisch.) S.B.Andrews	Polypodiaceae	FC505	Ind	metuapua'a	24	Restlessness, irritability, jerk	Leaf/Rhizome	Boil/Boil (in mixture)	Bath/Bath, massage, water spraying/Local application/Oral	11
						Fracture	Leaf	Boil (in mixture)/Crush (in mixture)	Bath/Local application/Oral	5
						Sinusitis	Rhizome	In mixture	ND	2
						Stomachache	Rhizome	Crush (in mixture)	Oral	2
						Teething	Leaf/Rhizome	Boil (in mixture)	Bath, local application/Oral	2
						Asthma	Rhizome	Crush (in mixture)	Oral	1
						Effects of reduced temperature	Leaf	Boil (in mixture)	Steam bath	1
						Epilepsy	Rhizome	Boil (in mixture)	Bath, local application	1
						Ranula (salivary cyst)	Leaf	Crush (in mixture)	Local application	1
						Skin disorders (he'a)	Leaf	Boil	Bath	1
*Morinda citrifolia* L	Rubiaceae	FC488	Ind	noni, nono	14	Ranula (salivary cyst)	Fruit (ripe)/Fruit (unripe)/Fruit (ripe), Fruit (unripe)	Crush/None	Local application/Oral	4
						COVID-19	Fruit (ripe)	Crush (in mixture)	Oral	3
						Detoxifying agent (puaroto)	Fruit (ripe)/Fruit (ripe), Fruit (unripe)	Crush (in mixture)	Oral	3
						Otitis	Fruit (ripe)/Fruit (unripe)	Crush (in mixture)	Local application/Oral	2
						Restlessness, irritability, jerk	Fruit (ripe)/Leaf	Boil (in mixture)/Crush (in mixture)	Bath/Oral	2
						Effects of reduced temperature	Fruit (ripe)	Crush (in mixture)	Oral	1
						Furuncles, abscess and others disorders with pus exudation	Leaf	Crush	Local application	1
						Headache	Leaf	Boil	Bath, steam bath	1
						Rectal prolapse	Leaf	Crush	Local application	1
						Skin disorders (due to mosquito bite)	Root	Crush (in mixture)	Local application	1
*Moringa oleifera* Lam	Moringaceae	NC	Mod	moringa	1	Fever	Flower, leaf	Crush	Oral	1
*Musa x paradisiaca* L	Musaceae	FC491	Mod	mei'a	2	Filariasis	Flower/Stem	Crush	Local application	1
						Sprain	Fruit (immature)	None	Local application	1
*Ocimum basilicum* L	Lamiaceae	FC543	Mod	miri	5	Restlessness, irritability, jerk	Aerial part/Leaf	Boil	Bath/Bath, oral	3
						Effects of reduced temperature	Aerial part	Boil	Steam bath	2
*Oxalis corniculata* L	Oxalidaceae	FC517	Pol	pātoa 'ava 'ava	2	Cough	Whole plant	Crush	Oral	1
						Fever	Whole plant	Crush	Local application	1
*Pandanus tectorius* var. *laevis* Warb	Pandanaceae	FC564	Pol	pae'ore	1	Skin disorders (he'a)	Shoot	Crush (in mixture)	Oral	1
*Paspalum vaginatum* Sw	Poaceae	FC553	Ind	matie tātahi	4	Asthma	Leafbud	Crush (in mixture)	Oral	1
						Cough	Aerial part	Crush (in mixture)	Oral	1
						Effects of reduced temperature	Aerial part	Boil	Steam bath	1
						Filariasis	Aerial part	Crush (in mixture)	Local application	1
*Persicaria glabra* (Willd.) M.Gómez	Polygonaceae	FC541	Ind	tamore, pītorea	9	Umbilical cord care	Aerial part/Leafbud/Stem/Stembud, leaf	Crush (in mixture)	Oral/Local application, oral	4
						Detoxifying agent (he'a)	Leafbud	Crush (in mixture)/Crush (in mixture), fermentation	Oral	2
						Lower abdominal disorders	Leaf/Leafbud	Crush (in mixture)	Oral	2
						Sprain	Leaf	Crush (in mixture)	Local application	1
*Phyllanthus tenellus* Roxb.*	Phyllanthaceae	FC532	Mod	moemoe	7	Furuncles, abscess and others disorders with pus exudation	Aerial part/Whole plant	Boil/Boil (in mixture)	Bath/Bath, massage, water spraying/Massage, water spraying	3
						Otitis	Leaf/Whole plant	Crush (in mixture)/Heat/Heat (in mixture)	Local application	3
						Restlessness, irritability, jerk	Whole plant	Boil	Bath	1
*Physalis angulata* L	Solanaceae	FC499	Pol	tāmaru ha'ari	1	Sinusitis	Leaf	Heat (in mixture)	Oral	1
*Premna serratifolia* L	Lamiaceae	FC548	Ind	’āvaro	1	Pain	Leaf	Boil	Bath	1
*Psidium guajava* L	Myrtaceae	FC567	Mod	tuava, tuvava	13	Restlessness, irritability, jerk	Fruit/Leaf/Leafbud	Boil/Boil (in mixture)	Bath/Bath, massage, oral/Oral	4
						Ranula (salivary cyst)	Leafbud	Crush (in mixture)	Local application/Oral	3
						Asthma	Fruit	Crush (in mixture)	Oral	1
						Detoxifying agent (he'a)	Bark, leafbud	Crush (in mixture), fermentation	Oral	1
						Diarrhea	Leafbud	Crush (in mixture)	Oral	1
						Fever	Leafbud	Boil (in mixture)	Oral	1
						Furuncles, abscess and others disorders with pus exudation	Leafbud	Chew/Crush	Local application	1
						Menstrual cycle bleeding disorders	Leaf	Boil (in mixture)	Oral	1
						Mouth ulcer	Fruit	Crush (in mixture)	Oral	1
						Otitis	Fruit (ripe)	Crush (in mixture)	Local application	1
						Scrotal swelling	Leaf	Boil (in mixture)	Bath	1
*Rorippa sarmentosa* (Sol. ex G.Forst. ex DC.) J.F.Macbr	Brassicaceae	FC530	Ind	pātoa pūrahi, moahau'a'ino	15	Ranula (salivary cyst)	Leaf/Whole plant	Crush/Crush (in mixture)	Local application/Oral	8
						Detoxifying agent (he'a)	Whole plant	Crush (in mixture)/Crush (in mixture), fermentation	Oral	2
						Tonsillitis, sore throat	Leaf/Whole plant	Crush/Crush (in mixture)	Gargling/Oral	2
						Asthma	Whole plant	Crush (in mixture)	Oral	1
						Cryptorchidism	Whole plant	Crush (in mixture)	Oral	1
						Vaginal and urethral discharge	Whole plant	Crush (in mixture)	Oral	1
						Yellow lip	Whole plant	Crush	Local application	1
*Rosa* sp.	Rosaceae	FC550	Mod	rōti 'ute'ute	7	Sinusitis	Flower	Crush with brown sugar	Local application/Local application, oral/Oral/Smell	7
*Saccharum officinarum* L	Poaceae	FC520	Pol	tō	33	Restlessness, irritability, jerk	Leaf/Leafbud/Stem	Boil (in mixture)/Crush (in mixture)	Bath/Bath, massage/Bath, massage, local application/Bath, massage, oral/Bath, massage, oral, water spraying/Bath, oral/Local application, massage, oral/Local application, oral/Massage, water spraying	19
						Sinusitis	Stem	Crush (in mixture)/Heat/Heat (in mixture)	Oral	7
						Lower abdominal disorders	Stem	Crush/Crush (in mixture)	Oral	6
						Skin disorders (he'a)	Stem	Boil (in mixture)/Crush (in mixture)	Oral/Local application, oral	5
						Umbilical cord care	Stem	Crush (in mixture)	Oral/Local application, oral	4
						Cough	Stem	Boil (in mixture)/Crush (in mixture)	Oral	2
						Detoxifying agent (he'a)	Stem	Crush (in mixture)/Crush (in mixture), fermentation	Oral	2
						Teething	Stem	Crush/Crush (in mixture)	Bath, oral, water spraying/Oral	2
						Bronchitis, productive cough	Stem	Crush (in mixture)	Oral	1
						Epilepsy	Stem	Crush (in mixture)	Oral	1
						Fever	Stem	Crush	Oral	1
						Flu	Stem	Crush (in mixture)	Oral	1
						Furuncles, abscess and others disorders with pus exudation	Leafbud	Boil (in mixture)	Massage, water spraying	1
						Otitis	Stem	Crush (in mixture)	Local application, oral	1
						Vaginal and urethral discharge	Stem	Crush (in mixture)	Oral	1
*Spondias dulcis* L	Anacardiaceae	FC501	Pol	vī tahiti	23	Cough	Fruit (unripe)/Leaf	Boil ( in mixture)/Crush/Crush (in mixture)	Bath, massage/Bath, oral/Gargling/Oral	12
						Tonsillitis, sore throat	Fruit (unripe)/Leaf	Boil (in mixture)/Crush	Bath, massage/Gargling/Gargling, oral/Oral	7
						Ranula (salivary cyst)	Fruit (unripe)	Crush/Crush (in mixture)	Oral	4
						Restlessness, irritability, jerk	Fruit/Leaf	Boil (in mixture)/Crush	Bath, massage/Bath, oral/Oral	3
						Asthma	Fruit	Crush	Oral	1
						Furuncles, abscess and others disorders with pus exudation	Leaf	Crush (in mixture)	Oral	1
*Syzygium malaccense* (L.) Merr. & L.M.Perry	Myrtaceae	FC529	Pol	’ahi'a	18	Detoxifying agent (he'a)	Leaf/Leaf, Leaf with gall	Crush (in mixture)	Oral	6
						Vaginal and urethral discharge	Leaf	Crush (in mixture)	Oral	5
						Skin disorders (he'a)	Leaf	Crush (in mixture)	Oral	2
						Asthma	Leaf	Crush (in mixture)	Oral	1
						Eczema	Leaf	Crush (in mixture)	Oral	1
						Flu	Leaf	Crush (in mixture)	Oral	1
						Menstrual cycle bleeding disorders	Leaf	Crush (in mixture)	Oral	1
						Mouth ulcer	Leaf	Crush	Gargling	1
						Urinary incontinence	Leaf with gall	Crush (in mixture)	Oral	1
						Vaginal prurit	Leaf with gall	Crush (in mixture)	Oral	1
*Terminalia catappa* L	Combretaceae	FC558	Mod	’autara'a	2	Acute blistering skin eruption	Leaf	Boil	Bath	1
						Filariasis	Leafbud	Crush (in mixture)	Bath, massage, oral	1
*Thespesia populnea* (L.) Sol. ex Corrêa	Malvaceae	FC508	Ind	miro	15	Fever	Fruit/Fruit (sap)/Fruit (unripe)	Crush (in mixture)/Cut and mix with water	Bath/Bath, local application/Oral/Oral, local application	4
						Furuncles, abscess and others disorders with pus exudation	Fruit	Boil/Crush (in mixture)	Bath/Oral	2
						Heat rash	Fruit (sap)	Cut and mix with water	Bath	2
						Chicken pox	Fruit (sap)	Cut and mix with water	Bath	1
						Detoxifying agent (puaroto)	Fruit	Crush (in mixture)	Oral	1
						Eczema	Fruit (sap)	Cut and mix with water	Bath	1
						Eye disorders	Fruit (sap)	Cut and mix with water	Local application	1
						Fracture	Fruit	Crush (in mixture)	Oral	1
						Headache	Fruit (sap)	Cut and mix with water	Local application	1
						Skin disorders (he'a)	Bark	Crush (in mixture)	Oral	1
						Skin disorders (due to mosquito bite)	Fruit (sap)	Cut and mix with water	Bath	1
*Torenia crustacea* (L.) Cham. & Schltdl	Linderniaceae	FC542	Pol	ha'eha'a, piriate	7	Lower abdominal disorders	Whole plant	Crush (in mixture)	Oral	2
						Sinusitis	Whole plant	Crush (in mixture)	Oral	2
						Detoxifying agent (he'a)	Whole plant	Crush (in mixture)	Oral	1
						Diarrhea	Whole plant	Crush (in mixture)	Oral	1
						Fracture	Whole plant	Crush (in mixture)	ND	1
						Restlessness, irritability, jerk	Whole plant	Crush (in mixture)	Oral	1
						Vaginal prurit	Whole plant	Crush (in mixture)	Oral	1
*Zingiber officinale* Roscoe	Zingiberaceae	FC499	Mod	re'a tinitō	5	COVID-19	Rhizome	Crush (in mixture)	Oral	3
						Detoxifying agent (he'a)	Rhizome	Crush (in mixture)	Oral	1
						Detoxifying agent (puaroto)	Rhizome	Crush (in mixture)	Oral	1
						Otitis	Rhizome	Crush (in mixture)/Heat, and crush (in mixture)	Oral	2
						Vaginal and urethral discharge	Rhizome	Crush (in mixture)	Oral	1

A slash (“/”) corresponds to the term “or”; a comma (“,”) correspond to the term “and”; Ind. = Indigenous; Mod. = Modern introduction; NC = Not Collected; ND = Not documented; Pol. = Polynesian introduction

^*^The plant called “moemoe” in Tahitian includes different species such as *Phyllanthus amarus*, *P. tenellus*, *P. virgatus*, and *P. urinaria*. In our study, we only collected *P. tenellus*

Of the reported 40 botanical families, Lamiaceae ranked first (7 species), Malvaceae ranked second (4 species), and Euphorbiaceae, Moraceae, Poaceae, Rubiaceae, and Zingiberaceae ranked third (3 species each). *Coleus* and *Hibiscus* were the most represented genera (3 species each), followed by *Allium*, *Citrus*, *Cyperus*, and *Ficus* (2 species each).

The most common used plant organs were leaf (39 species), followed by leafbud (15 species), fruit (14 species), flower (8 species), whole plant (8 species), and bark (7 species). In terms of citations, leaf ranked first again (266 UR), followed by fruit (163 UR), leafbud (58 UR), stem (54 UR), rhizome (44 UR), and whole plant (39 UR). The importance of leaves and fruits in the Polynesian pharmacopeia can be partly explained by their ease of access and also by the fact that it lends itself perfectly to the method of preparation expressing the juice of the plant by pressure.

**Fig. 2:**
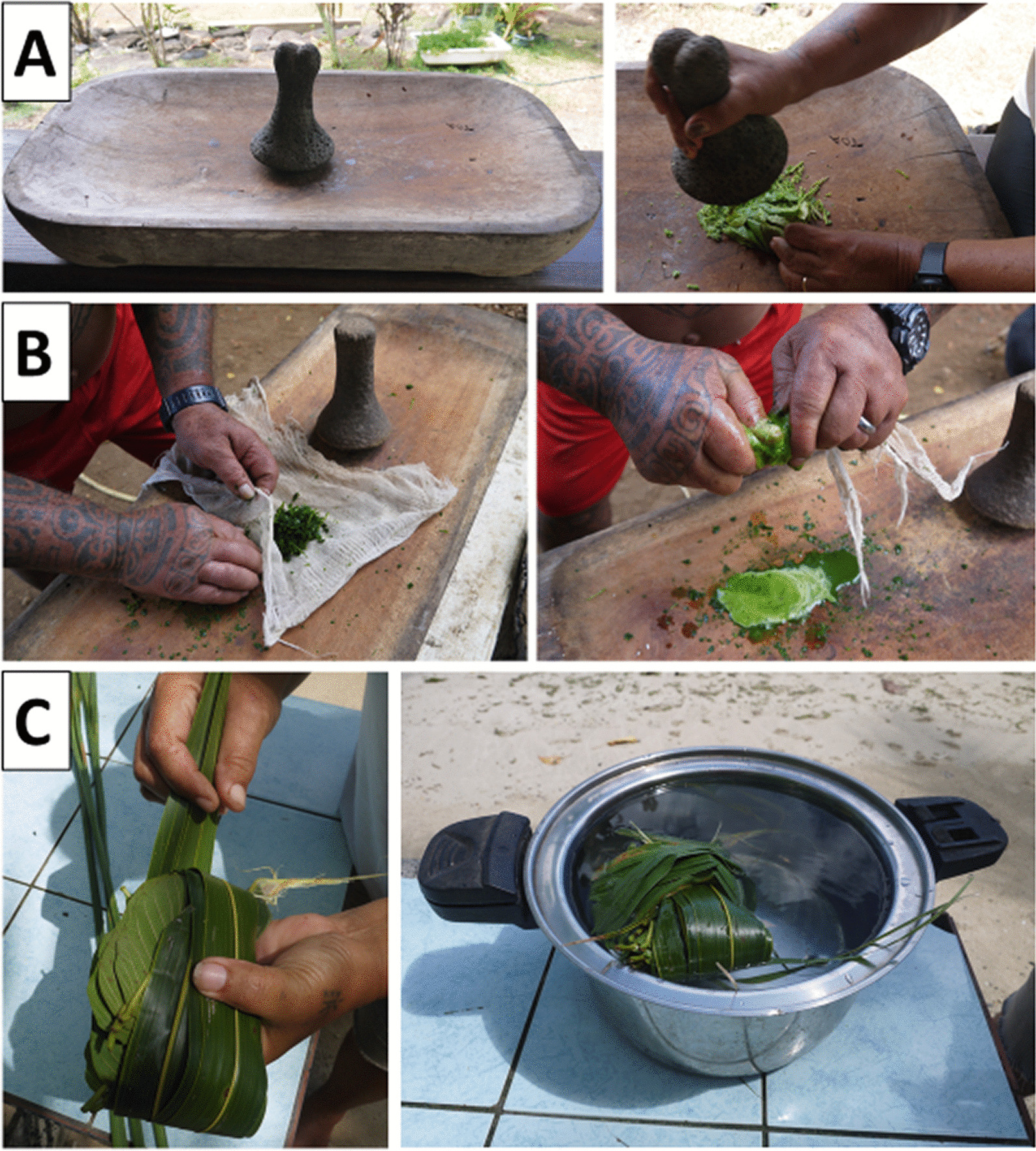
Main methods of preparation used in Polynesian traditional medicine as reported by the 86 participants from the five islands in the Society archipelago. **A** Mortar and pestle used to crush the ingredients; **B** Cotton cloth used to express the juice from the crushed ingredients; and **C** Method of assembling herbal ingredients before putting them in boiling water for decoction

The most common methods of preparation reported were, respectively: to crush and press the plant(s) in a cloth to get the juice (52 species), to boil the plant(s) in water (37 species), to crush the plant(s) and let it/them ferment for a few days (12 species), to heat the plant(s) (7 species), to use the plant(s) without any preparation (6 species). In terms of citations, crushing and pressing the plant(s) in a cloth to get the juice ranked first (441 UR), boiling the plant(s) in water ranked second (231 UR), crushing the plant(s) and let it/them ferment for a few days ranked third (15 UR), cooking the plant(s) ranked fourth (14 UR), and using the plant(s) without any preparations ranked fifth (13 UR). Previous studies already reported the importance of crushing the plant and getting the juice by expression in the Polynesian pharmacopeia [2, 4, 5, 14]. This practice is strongly associated with the widespread use of tools (i.e., mortar and pestle) in Polynesian cuisine (Fig. 2). Here, these utensils are employed to crush the plants. Regarding the expression of juice, it can be realized with homemade fiber cloths made either from *Cyperus javanicus* stems or from coconut fluff [14]. Nowadays, these homemade fiber cloths have been mainly replaced by a commercialized cotton cloth.

The most common methods of administration mentioned were oral (48 species), local application (34 species), bath (28 species), bath and oral (12 species), and bath and massage (10 species). In terms of citations, oral administration ranked first (370 UR), followed by bath (121 UR), local application (86 UR), bath and massage (23 UR), and bath and oral (20 UR). Although oral administration and massage are largely described in the Polynesian literature [1, 2, 5], taking a bath was not so common in adults. Therefore, we can hypothesize that this method of administration is mainly used for babies and children.

Of the 469 reported remedies, 223 (47.5%) were administered for 3 days, 11 (2.3%) were advised to be taken until healing, 5 (1.1%) until the remedy is finished, 3 were administered for 7 days, 3 others were administered for 1 day, and 3 others were administered as much as the patient wanted. For 212 (45.2%) of the remedies, no duration of intake was reported. The 3-day rules of administration are one of the most remarkable specificity of Polynesian pharmacopeia [14, 22]. However, it does not seem to be limited to the French Polynesian islands as this practice was also described in the Cook Islands [20]. This could be explained by a link to the Christian doctrine of the Trinity, albeit it is difficult to ascertain this information [5].

Regarding their biogeographical origin, 28 (41.8%) plant species were introduced by Europeans and other population (Chinese, Japanese, Polynesians) after eighteenth century, 21 (31.3%) plant species were introduced in the islands by Polynesians (during their migration from the Western Pacific at the end of the first millennium), and 18 (26.9%) can be classified as indigenous. This is another specificity of the Polynesian pharmacopeia, most of the plants used are introduced species. Other publications already reported this information [1, 2, 5, 23].

#### Zootherapy

In this study, nine ingredients from animals were mentioned by the participants representing a total of 24 UR (Table 4). The most cited zootherapy ingredients was the soft tissue from the stone urchin (5 participants, 5 UR), followed by honeybee (4 participants, 6 UR), and venom from bees (3 participants, 3 UR). The most represented method of preparation was cooking the ingredients to get the oily phase (5 participants, 9 UR). The most represented method of administration was local application (12 participants, 10 UR). Sinusitis was the most reported health disorder treated by zootherapy ingredients (6 participants, 10 UR) followed by cough (4 participants, 4 UR) and otitis (3 participants, 3 UR).

**Table 4:** Zootherapy ingredients reported in the survey by the 86 participants from the five islands in the Society archipelago

English name	Scientific name	Tahitian name	Part used	Type of disorders treated	Method of preparation	Method of administration	Number of persons citing the ingredient	Number of UR
Domesticated bee	*Apis mellifera*	manu meri	venom	Effects of reduced temperature, epilepsy, headache, otitis, respiratory disorders, rheumatism, sinusitis	None	Local application	3	3
Domesticated bee	*Apis mellifera*	manu meri	honey	Asthma, COVID-19, **cough**	In mixture	Oral	4	6
Stone urchin	*Echinometra mathaei*	’ina	soft tissue	Furuncles, abscess, and other disorders with pus exudation, skin disorders, **sinusitis**	Crush, cook (in mixture)	Local application/Oral	5	5
Domesticated chicken	*Gallus gallus*	moa	buttocks	Sinusitis	Cook, get the oil	Local application	1	1
Domesticated chicken	*Gallus gallus*	moa	liver	Sinusitis	Cook, get the oil	Local application	1	1
Human	*Homo sapiens*	ū māmā	breastmilk	Conjunctivitis	None	Local application	2	2
Crab	NI	tota	soft tissue	Sinusitis	Cook (in mixture)	Oral	1	1
Centipede	*Scolopendra subspinipes*	veri	whole part	Otitis	In mixture with monoi	Local application	2	2
Common fiddler crab	*Uca tetragonon*	’aramihi	soft tissue	Furuncles, abscess, and other disorders with pus exudation, **sinusitis**	Cook (in mixture)	Local application/Oral	2	3

Legend: A slash (“/”) corresponds to the term “or”; a comma (“,”) corresponds to the term “and”; NI = Not Identified; in bold are the most cited ailments for each ingredient

As previously discussed [24], the use of animal species in medicine can raise concerns about illegal and unethical uses. In this study, none of the species cited by participants have been reported by the CITES (Convention on International Trade in Endangered Species of wild fauna and flora) or the IUCN (International Union for Conservation of Nature) as endangered or vulnerable species. Moreover, none of these species belong to the category A or B for protected species established by the Environment Department in French Polynesia. Also, stone urchin (*Echinometra mathaei*) has been described as a common and widely distributed species in the Indo-Pacific [25].

#### Other ingredients

Besides the use of plants and animals, other ingredients were employed by participants to prepare their remedies. Among them, monoi was the most cited ingredient (19 participants, 23 UR). In all cases, monoi was applied locally, including in massage (4 cases). Other mentioned ingredients included: mint alcohol (2 part. 2 UR), seawater (2 part., 2 UR), sugar alone (2 part., 2 UR), salt (1 part., 1 UR), starch (1 part., 1 UR), and white vinegar (1 part., 1 UR).

Monoi is a very popular cosmetic product in French Polynesia, and it is widely marketed throughout the world as an iconic Polynesian product. Monoi is made from coconut oil mixed with perfumed flowers or leaves, especially flowerbuds or open flowers from *Gardenia taitensis*. It is mainly employed for body and hair care [23]. Its use in medicine is less known, but it represents a significant part of the Polynesian pharmacopeia. In our study, monoi was reported for treating mainly otitis (7 participants) and fracture and sprain (6 participants). In most cases, monoi was cited to be prepared in mixture with other plant species and applied locally. Other studies already reported the use of monoi in medicine in French Polynesia for treating: otorrhea (ear discharge), fracture and sprain, furuncles and abscesses, and for backache and prick from rusted nails [4, 14, 26]. Of note, Pétard indicated that newborn babies in French Polynesia are regularly massaged with monoi from head to toe during their first months [4].

#### Other Polynesian medical practices

As mentioned in the overview of Polynesian medical practices section, manipulative practices were also reported by participants. This includes massage (39 participants), blowing into the mouth (8 participants), pushing on the gingiva (4 participants), and cutting the gingiva (3 participants). Massage therapy was mentioned as an adjuvant of a treatment. While we did not record the disorders treated by massage, we recorded them for the three other practices. Blowing into the mouth was reported to be used for sinusitis. It consists of exhaling air through the mouth of a child and by blocking his/her nose at the same time. It is said to help decongest the sinuses. Pushing the gingiva (defined as pushing hard on the gingiva with a finger) and cutting the gingiva were used to treat teething disorders and especially helped the teeth come out.

The act of treating teething by cutting the gums with a lancet is an old medical tradition that was first scientifically developed by a French army surgeon, named Ambroise Paré, in 1575. This method was later used by English doctors, and so until the middle of the twentieth century [27]. Because English and French deeply influenced the Polynesian medicine, we can suggest that this practice of lancing gingiva might have been introduced by French and English medical doctors many decades ago. Although cutting gingiva might have been rooted in biomedical medicine, nowadays this practice is no more recommended by health professionals, especially as it can induce severe complications [28].

#### Other aspects of Polynesian medicine

During the survey, some participants provided additional information regarding the use of traditional medicine in general. Here, we give details regarding this information.

First of all, participants mentioned general rules of administration. For two participants, remedies have to be taken before 3 or 4 PM. Another participant also reported not giving a remedy in the afternoon. They think that the remedies might not be effective if this rule is not respected.

Secondly, three participants noted that plants or remedies already used should be discarded in specific places and not in the regular trash can. These places include homegardens (*Fa’a’apu*), flowerpots, and any locations where the plant can return to the earth.

Thirdly, food restrictions were also reported by participants. When the children is sick, meat, oil, and salt should be avoided (one participant). In the same way, another participant mentioned that chips, cheese, iced water, and iced juice are not recommended.

#### Dangerous practices reported by participants

One of the questions asked during the interviews focused on the dangerous traditional practices or treatments known by the participants. To this question, 45 participants (52.3%) mentioned the plant “metuapua'a” (*Microsorum grossum*), 11 participants (12.8%) answered the plant “ti'ati'amou'a” or “titi” (*Davallia solida*), 5 participants (5.8%) cited “tiare tahiti” (*Gardenia taitensis*), 3 participants mentioned “pātoa” (*Rorippa sarmentosa*), 3 participants expressed concerns about a dosage not respected, 2 participants mentioned the practice of blowing air into the children’s mouth, 2 participants cited avoiding combining conventional treatments and traditional treatments, 2 participants mentioned the plant “noni” (*Morinda citrifolia*), and 2 participants cited chili pepper (*Capsicum frutescens*). Finally, 23 participants did not answer this question. Regarding the plant “metuapua'a,” some participants provided details on the source of danger when using this plant. Of the 45 participants mentioning “metuapua'a,” 10 cited roots as being more dangerous, 7 participants mentioned the use of wrong dosage, 6 participants cited the oral administration, 6 participants mentioned avoiding combining this plant with conventional treatment, 6 participants reported that children are more prone to side effects than adults, 6 participants cited that the plant can induce death, and 4 participants mentioned that the plant should not be used at the same time than vaccinations.

The plant species called “metuapua'a” was already reported in other ethnobotanical studies as being widely used in French Polynesia: for fracture, shock, fall in the Marquesas islands [2], as a purgative and vermifuge in children [4], and as one of the most used fern species in Polynesian medicine and the Pacific [29]. In the book of Pétard, it was reported that this fern is mainly used as a purgative and vermifuge in children [4]. Besides its popularity, many reports of its toxic potential have been documented. From 1967 to 1970, various poisonings due to “metuapua'a” were reported in newborn babies and children [4]. In Moorea, different traditional healers mentioned *Microsorum grossum* as being poisonous [22, 30]. The medicinal potential of *Microsorum* species was attributed to the presence of phytoecdysteroids [31]. These compounds possess steroid-like effect and are recognized as safe [32, 33]. Therefore, the toxicity observed when using species from the genus *Microsorum* is still controversial. Some authors proposed that botanical confusion between different fern species may cause this toxicity [29]. This hypothesis is supported by different studies showing that the genus *Microsorum* is highly confusing from a taxonomic point of view [34, 35]. We can also state that other compounds present in the fern species could be responsible for its toxicity. Because most of the studies on *Microsorum* involved the use of leaves (fronds), roots that are less studied could contain such toxic compounds not already described. Also, phytochemical variation following temperature and seasonal variation, geographical location, or time of collect could also be involved. Of note, “metuapua'a” is also widely mentioned to be combined with other plant species especially “ti'ati'amou'a” [4]. In our survey, this plant species is also reported as the second most poisonous species. This suggest that compounds interactions between fern species could also be involved.

### Types of health disorders and therapeutic management

Of the 69 health disorders cited, some had a high proportion of similar used plant species, thus showing cultural consensus among participants. This feature was identified thanks to the ICF index (Additional file 1: Table S1). The set of disorders called *ira* showed the highest ICF with 0.86 (158 UR, 23 used plant species), followed by tonsillitis and sore throat with an ICF of 0.8 (11 UR, 3 plant species), the set of disorders called *he’a* with an ICF of 0.78 (124 UR, 28 plant species), chickenpox with an ICF of 0.73 (12 UR, 4 plant species), vaginal and urethral discharge with an ICF of 0.7 (24 UR, 8 plant species), and ranula (salivary cyst) with an ICF of 0.68 (29 UR, 10 plant species). In terms of disease categories, mental, behavioral or neurodevelopmental disorders ranked first with an ICF of 0.86 (158 UR, 23 plant species), digestive system ranked second with an ICF of 0.76 (97 UR, 24 plant species), respiratory system ranked third with an ICF of 0.74 (125 UR, 33 plant species), genitourinary system ranked fourth (*ex-aequo*) with an ICF of 0.68 (48 UR, 16 plant species), and skin disorders ranked fourth (*ex-aequo*) with an ICF of 0.68 (77 UR, 25 plant species).

Among cited plant species, some were mostly dedicated to the treatment of specific disorders. This feature was identified thanks to the FL index (Additional file 2: Table S2). *Annona muricata* had a Fidelity Level (FL) of 100% for *ira*, *Rosa* sp. had a FL of 100% for sinusitis, *Hibiscus rosa-sinensis* “Carnation” had a FL of 87.9% for *ira*, *Syzygium malaccense* had a FL of 83.3% for *he'a*, and *Curcuma longa* had a FL of 82.8% for *he'a*. In terms of disease categories, *Coleus scutellarioides* had a FL of 100% for injury (sprain, fracture), *Spondias dulcis* had a FL of 78.3% for respiratory disorders (cough, tonsillitis, sore throat, asthma), and *Ficus tinctoria* had a FL of 71.4% for skin disorders (skin disorders related to *he’a*, eczema, furuncles, abscess, and other disorders with pus exudation).

In the following sections, we present in descending order of importance, the results obtained for each children disease cited by more than ten participants. In the section detailing each reported remedy, only data for remedies cited by three participants and more are presented.

An assessment of the efficacy and toxicity (based on a thorough bibliographic review) was performed for each most cited remedy. A summary of these evaluations can be found in Additional file 3: Table S3.

In Table 5, we detail the information on symptoms, causes, methods of diagnosis, and methods of prevention gathered from the participants to this survey for the most cited children illnesses.

**Table 5:** Description of the most cited children illnesses by the 86 participants in the five islands from the Society archipelago

Disorders	Number of informants	Number of remedies	Number of UR	Symptoms	Age	Causes	Method for diagnosis	Prevention	Evolution
Restlessness, irritability, jerk (ira*)	60	50	95	Jerk (21) Blue spots on the skin (7) Epileptic symptoms (5) Eyes with specific color (5) Restless baby (5) Green feces (2)	From birth (5) > 3 months old (2) > 1 month old (1) 0–10 years old (1)	Mothers (4): bad things said in the past (1), jerk while watching TV (1), Lack of cleanliness (1)	Presence of blue or dark spots on the skin or around the eyes (4)	Use remedy for ira (8): every month (2), every 15 days (1), every 3 months (1), for children up to 2 years old (2)	ND
Sinusitis (nanu*)	35	30	39	Mucus in the nose (6) Blocked-up nose (4) Running nose (3) Breathing difficulties (2)	> 3 months old (3) From birth (2)	Disorders related to delivery (3) Cold not well treated (2) Catching cold (2) Allergy (1) Lack of cleanliness (1)	ND	Protecting babies from cold temperature (1) Protecting babies from sun and hot temperature (1)	Can develop into a more serious health condition, e.g., bronchitis, otitis, regurgitation (4)
Ranula (salivary cyst) (arero ma'a*/*double tongue*)	25	17	27	Piece of flesh underneath the tongue (14) Tonsillitis (8) Difficulty in swallowing (3) Red throat (3) Cough (2) Sore throat (2) Swollen tongue (2) Fever (1) Hard jaw (1) Presence of mucus (1)	ND	Burns caused by hot meals (11) Teething (3) Genetic background (1) Lack of hydration (1) Microbes (1)	ND	Avoid drinking hot water (1)	Can develop into an acute rheumatic fever (1) Can lead to coma (1)
Teething (ira niho, niho*)	24	10	17	ND	ND	ND	ND	ND	ND
Cough (hota*)	23	15	25	ND	ND	Catching a cold, weather too cold (3) Children's relatives (smoke, suffer from a cold themselves) (2) Sea, rivers, rain (1)	ND	ND	Dry at the beginning, then develop into a productive cough (1)
Fracture (fati*)	18	14	21	ND	ND	Accident (3) When a parent does not hold a baby carefully (3)	Baby cries after touching his backbone (1) Cold feet, red lips, fever (1)	ND	ND
Fever (fiva*)	17	14	19	ND	ND	ND	ND	ND	ND
Detoxifying agent (*he'a*, pu'a roto*)	14	13	15	Please refer to the description of *he'a* in the text					
Vaginal or urethral discharge (he'a*)	14	7	11	Vaginal and urethral discharge (11) Genital itching (3) Genital odors (1) Painful urination (1)	> 5–6 years old (1) 4–8 years old and teenagers (1)	Unhealthy food, bad breath, running nose, eye gunk, and because the mother was not treated (with a rā’au he’a) during her childhood and adulthood (1)	ND	Please refer to the description of he'a in the text	Can develop into a cervical cancer (1)
Asthma (ahopau*)	13	11	11	Breathing difficulties (6): air hunger (1), fever (1) Occurs occasionally (1)	Start as soon as the child walks (1)	Allergy (1) Dust (1) Genetic (1) Injury (1) Lack of health education (1) Lack of medical equipment (thermometer) (1) Pollen (1) Tobacco (1) Untreated wound (1)	Visual inspection and take the pulse (1)	ND	ND
Furuncles, abscess, and other disorders with pus exudation (tui*)	13	11	12	Furuncles localized on the head (4) Furuncles spreads all over the body (2) Pain (1) Pus exudation (1) Stick eyelids (1)	ND	“staphylo” (1)	ND	ND	ND
Otitis (*tari'a ma'i*, tui*)	13	13	15	ND	> 3 months old (1) Babies up to 1 year (1)	Take a bath or swimming (4) Diving deep into the sea (1) Running nose, cold poorly treated (1)	ND	Do not dive into the sea (1)	ND
Lower abdominal disorders (tia*)	12	13	13	Lower abdominal pain (6) Difficulty urinating (5) Lower abdominal tightening (4) Pain on urination (3) Colic (1) Constipation (1) Fever (1) Hamstring pain (1)	Boys and girls from birth (1) > 1 month old (1) > 2 years old (1)	Poorly healed belly button (1) Food or milk (1)	Palpation of the child's belly (1)	Drink a lot of water during the day (1)	ND
Skin disorders (associated with *he'a*)	12	11	11	Itching (2) Blisters (1) Microbial skin infections (1) Pimples (1) Spots (1) Localized in the wrinkles (1) Spread all over the body (1)	ND	Lack of treatment (with ra'au he'a) for “cleaning” the babies and pregnant mothers (3)	ND	Take the remedy “ra'au he'a” during pregnancy and childhood (3) Avoid sugar, soda, and food in cans (1)	Does not heal (1)
Umbilical cord care (pito*)	12	7	8	Abdominal pain (1)	From 1 month to 1 year old (1) 1 month old (1) 3 months old (1) Newborn babies (1)	Poor healing of the navel in newborn babies (6) Green feces stuck in the intestine (1) Running nose (1) Cold (1)	ND	Place a bandage on the navel (3). This avoids cold to penetrate inside the body	ND
Chickenpox ('ōniho*)	11	5	13	Blisters (2) Red spots (1) Staphylococcus (1)	> 2–3 years old (1)	Sun allergy (1)			Risk of outbreak, contagious (2)
Effects of reduced temperature (puta to'eto'e*)	11	7	9	Shivers (3) Cold body (2) Backache (1) Bronchitis (1) Cold sweat (1) Colic (1) Flu (1) Headache (1)	ND	Umbilic not protected from cold temperature (3) Cold temperature (2) Seawater bath, sweating, walking on cold surfaces (such as tiles) (1) Unbalanced body (1)	Feel cold when touching the palm of a child's hand	Be careful when taking a bath (1)	ND

* = Tahitian name; ND = Not Documented; number of informants are indicated in brackets; UR = Use-Reports

#### Ira

As stated above, the set of disorders called “ira” can be defined as a group of behavioral disorders including restlessness, irritability, to wake with a start, and convulsion. This definition was established based on information provided in our study and previous data on this topic.

A total of 50 unique remedies were cited for treating *ira* corresponding to 95 UR. Up to seven ingredients were combined in the remedies (see also Additional file 4: Table S4 for a detail on the number of ingredients present per remedy). Thirteen plant species belonging to 11 families were reported to be used to treat *ira*. The most cited plant species used for treating *ira* were *Annona muricata* [leaf] (33 UR), *Hibiscus rosa-sinensis* “Carnation” [flower or leaf] (29 UR), *Gardenia taitensis* [flowerbud or leaf] (28 UR), *Saccharum officinarum* [leaf, leafbud, or stem] (19 UR), and *Microsorum grossum* [leaf or rhizome] (11 UR).

The most cited remedy (21 participants, 33 UR) was composed of one ingredient: the leaves of *Annona muricata*. This preparation is boiled in water (20 participants, 95%) and mainly used (20 participants, 95%) for bathing the babies and children. In addition to the bath, five (23.8%) participants also added massages, five (23.8%) administered orally one or two teaspoons of the decoction, three applied the decoction locally on the fontanel, and two sprayed the decoction on the child’s face. Five participants reported using 30 leaves, four participants use 1 full hand of leaves, and two participants use about 12 to 15 leaves. Eight (38.1%) participants give the remedy for 3 days, and five (23.8%) participants use it once a day. This remedy was cited as having a sedative effect on children by two participants. It was also cited to dry out the skin (1 participant), clean up the body (1 participant), and soothe the bath (1 participant).

##### Ethnobotanical, pharmacological, and clinical data on this remedy (see Additional file 3: Table S3 for a summary)

In the scientific literature, *A. muricata* leaves were already reported to be used as a sedative in the Caribbean, in the Guianas, in Martinique, in Peru, in West Africa, and in the West Indies. Other related ethnobotanical uses included anxiety, anticonvulsant, antispasmodic, hypotensive, and smooth muscle relaxant [36]. In Suriname, a bath with a decoction of *A. muricata* leaves was used in children to decrease crying [37]. The anxiolytic effect of an alkaloid fraction obtained from an hydroalcoholic extract from the leaves of *A. muricata* was demonstrated in a mice model by using the elevated plus-maze test [38]. Although the mechanism of action is not completely elucidated, it could involve the GABAergic, and/or the monoaminergic (e.g., via serotonin, dopamine) systems [39]. Regarding the compounds responsible for the activity, isoquinoline-type alkaloids such as anonaine, asimilobine, and nornuciferine have been shown to bind 5-HT_1A_ receptors (serotonin receptors that are involved in the modulation of emotion) [40]. Interestingly, other *Annona species* (i.e., *A. cherimolia* and *A. coriacea*) have also demonstrated anxiolytic-like effects in mice. In the case of *A. cherimola*, the GABA_A_ receptor has been shown to be involved in this activity, and compounds such as palmitone have been suggested to be responsible for this activity [41, 42]. It is worth mentioning that the chronic consumption of *A. muricata* fruits and herbal tea of *A. muricata* leaves was associated with atypical parkinsonism in the Caribbean [43]. Acetogenins (especially annonacin) along with isoquinoline alkaloids are highly suspected to be in cause in this neurotoxicity [44, 45]. Clinical trials have been performed in adults with diabetes and colorectal cancer, and the safety was confirmed for an oral dose up to 540 mg/day of *A. muricata* leaf extract when taken for up to 30 days [46]. Overall, *A. muricata* leaves present ethnobotanical and pharmacological evidence of efficacy as sedative agent. However, using the plant might induce neurotoxicity. It is unlikely that such phenomenon happens when using the decoction of *A. muricata* leaves occasionally and externally (bath), but users should be aware that taking orally *A. muricata* leaves or fruits everyday for a long time might induce toxicity.

The second most cited remedy (11 participants) was composed of two ingredients: the leaves of *Hibiscus rosa-sinensis* “Carnation” and the leaves of *Gardenia taitensis*. Of the 11 participants, six mentioned combining these two plants with sugarcane leaves. All these preparations are boiled in water (11 participants, 100%) and mainly used for bathing the babies and children (10 participants, 91%). Only one participant mentioned to combine a local application and an oral administration of the decoction. In addition to the bath, four participants also mentioned to administer one or two teaspoons of the decoction orally, three massage the babies and children, two sprayed the decoction on the child’s face, and one applied the decoction locally on the skin. Seven participants (63.6%) reported collecting 30 leaves of the two plant species, one participant mentioned using from 20 to 30 leaves, another one reported employing 15 leaves, and the last one mentioned 5 leaves (one participant did not answer this question). For the six participants adding sugarcane to the remedy, two reported using 3 leaves, two other reported using 2 leaves, and one mentioned using 1 leaf only. Five (45.4%) participants dispense the remedy for 3 days. Of note, the leaves of *Gardenia taitensis* and the leaves of *Hibiscus rosa-sinensis* “Carnation” were also cited to be used separately (2 participants each) for bathing the babies and children. Two other participants reported to use the flowers of *Hibiscus rosa-sinensis* “Carnation” instead of the leaves. And finally, the leaves of *Gardenia taitensis* and the leaves of *Hibiscus rosa-sinensis* “Carnation” were also cited to be combined with *Annona muricata* leaves by two participants.

##### Ethnobotanical, pharmacological, and clinical data on this remedy

In the Polynesian literature, *H. rosa-sinensis*^1^ leaves were already reported to be used for *ira* either alone in the Marquesas Islands and in the Cook Islands [2, 48], or in mixture with *G. taitensis* leaves in the Cook Islands [20]. In Tahiti, *G. taitensis* leaves were reported to be used in mixture with *Broussonetia papyrifera* (L.) Vent. and sugarcane for treating *ira ‘īriti* [a type of *ira*] in children [14]. In the Cook Islands, *G. taitensis* flowers were mentioned for treating *ira* in children [20]. In other countries, the leaves of *H. rosa-sinensis* were employed as a sedative in Unani medicine, and for child sleeplessness in the Philippines [49]. Regarding its pharmacological activities, none of the properties associated with its Polynesian ethnobotanical uses were studied on leaves. However, other parts of the plant showed interesting activities. Roots of *H. rosa-sinensis* have been proven to possess anxiolytic effect in mice via GABAergic actions [50]. Flowers of *H. rosa-sinensis* have been shown to be anticonvulsant in mice, and their activities on the GABA_A_ receptor were also suggested [51]. So far, no compounds responsible for these activities have been isolated. Toxicity of *H. rosa-sinensis* leaves was evaluated in mice, and it was not toxic to a single oral dose of 2000 mg/kg, but sub-acute toxicity at 800 mg/kg for 14 days induced hepato-renal toxicity, thus indicating that low dose of *H. rosa-sinensis* should be used [52]. Regarding *G. taitensis*, no data are available on its pharmacological activities, but over 150 volatile compounds have been isolated from the flowerbuds including salicylate derivatives (compounds with anti-inflammatory and analgesic activities). Overall, *H. rosa-sinensis* leaves and *G. taitensis* leaves present ethnobotanical evidence of efficacy, but pharmacological and phytochemical studies are lacking and more researches should be performed to validate the use of leaves especially in children.

The third most cited remedy (6 participants) was composed of one ingredient: the leaves (also called fronds) of *Microsorum grossum*. This remedy is boiled in water (6 participants, 100%) and used for bathing (5 participants, 83.3%) or taken orally (1 participant). Of the six participants, one mentioned to apply the decoction on the fontanel in addition to use it for bathing. No real consensus was found on the number of leaves to be used in the remedy. One participant uses 3 leaves, another one 6 leaves, a third one mentioned using 10 leaves, a fourth one uses 20 leaves, and the last one mentioned a full hand of leaves. However, all participants agreed on using it for 3 days from 1 to 3 times a day.

##### Ethnobotanical, pharmacological, and clinical data on this remedy

Similar ethnobotanical uses were reported in other Polynesian countries. In Samoa, *M. grossum* leaves or rhizomes are used to treat childhood diseases such as *ila mea* and *ila fale* [53]. In the Cook Islands, *M. grossum* leaves or rhizomes are used to treat *ira* [20]. As mentioned previously, *M. grossum* contains phytoecdysteroids such as ecdysone and 20-hydroxyecdysone that possess steroid-like effect. However, the frequent reports of toxic events, especially in children, make the plant unsafe to use internally in children.

The fourth most cited remedy (4 participants) was made of one ingredient: the leaves of *Cordyline fruticosa*. This remedy is rubbed (2 participants) or boiled (1 participant) in water and then used for bathing (3 participants). In addition to the bath, two participants also massage the babies and children, one gives the remedy orally, and another one sprays the water on the child’s face. Of the four participants mentioning this remedy, only two indicated the posology. They administer the remedy for three days.

##### Ethnobotanical, pharmacological, and clinical data on this remedy

*C. fruticosa* has a high importance in the Polynesian culture, as it is used for many purposes, i.e., clothing, ornamental, food, alcohol preparation, rituals such as walks on fire, and religious ceremonies [54, 55]. This plant is also employed for treating various ailments in Hawaii, the Cook Islands, the Marquesas islands, Samoa, and the Society Islands [4, 20, 53, 56]. Although no record of the plant for treating *ira* was found in these publications, leaves dipped in water were already reported to be used for general massage and aches in Samoa, and for fever in Hawaii. *C. fruticosa* leaves have been poorly studied from a pharmacological view, but their importance in Polynesian culture tends to indicate that their healing power might be based on their spiritual role (albeit further studies are needed to confirm this hypothesis).

**Fig. 3:**
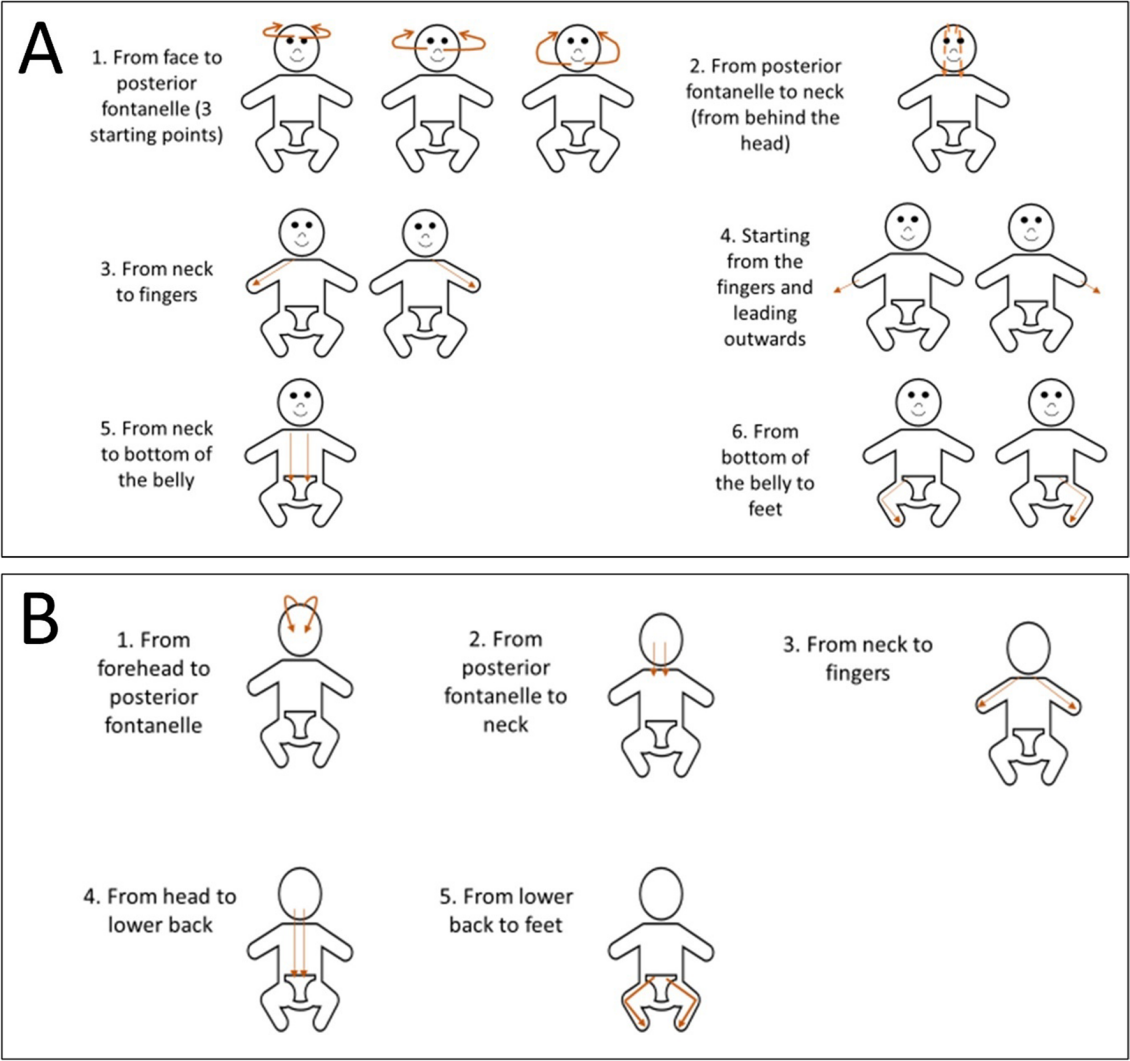
Methods used to massage infants and children with “ira” as reported by three participants from the Society archipelago. Legend: **A** Front massage. **B** Back massage

In addition to the use of traditional remedies, massage therapy was often employed for treating *ira*. During our survey, we noticed that the methods used to massage the babies and children shared some common points between participants. We thus decided to ask three participants (one specialist from Tahiti and expert in the treatment of childhood diseases, one specialist from Bora Bora and expert in herbalism and massage therapy, and one non-specialist from Bora Bora) to explain us this method. The objective of this therapy was said to help the flow of energy throughout the body and expel the energies that need to be expelled. To induce the movement of energy, a pressure is applied on the body by using thumbs, while a movement from one part of the body to another is made. This practice is sequential and present different steps (Fig. 3). Each step is repeated several times, and then, the energy is moved step by step to the extremities (fingers and toes) where the energy is expelled from the body. To achieve this, each finger and toe are pinched and pressed outward before releasing it. Each massage starts from the head (i.e., face and fontanel) that are considered as focal points. In total, the massage can last from 30 to 90 min, and one of the remedies cited below can be applied to the skin and used to perform the massage. At the end, the infants or children are coated with monoi and covered with a towel in a draught-free place.

#### Sinusitis

Sinusitis was mentioned by 35 participants and represented 39 UR. The Tahitian name employed to identify sinusitis was “nanu.”

A total of 30 unique remedies were cited for treating *nanu*. From 1 to 7 ingredients were combined in the remedies. In these remedies, 20 plant species belonging to 16 botanical families were reported to be used for treating *nanu*. The most cited plant species were *Cocos nucifera* [coconut milk or coconut water] (11 UR), *Rosa* sp. [flower] (7 UR), *Saccharum officinarum* [stem] (7 UR), *Cordyline fruticosa* [leaf or leafbud] (3 UR), and *Leucas decemdentata* [aerial part, stem, or whole plant] (3 UR).

The most cited remedy (7 participants, 20%) was composed of *Rosa* sp. flowers mixed with brown sugar. This remedy was crushed, then put in a cloth, and pressed to get the juice (7 participants, 100%). It was either taken orally (3 participants), applied locally (2 participants), smelled (1 participant), or taken orally and applied locally (1 participant). Four participants mentioned using this remedy for 3 days. One participant mentioned using 1 flower, another one mentioned mixing 3 flowers with 1 teaspoon of brown sugar, a third one mentioned using 1 to 3 flowers, and the last one reported mixing 6 flowers with 1 teaspoon of brown sugar.

##### Ethnobotanical, pharmacological, and clinical data on this remedy

Flowers from *Rosa* species were already reported to be used in Raiatea and Tahaa for treating sinusitis [3]. Apart from this reference, we did not find any other ethnobotanical records for this plant in the Polynesian literature, suggesting that the use of *Rosa* species in Polynesian medicine is quite recent and/or limited to some islands (i.e., Bora Bora, Raiatea, Tahaa). In Bolivia, the flowers of *Rosa canina* are used for treating sinusitis [57]. In Spain, *Rosa canina* flowers are also used for treating catarrh [58]. In Turkey, various *Rosa* species are used in medicine, especially for the treatment of colds, cough, sore throat [59]. The use of *Rosa* species for respiratory tract disorders could be attributed to the presence of vitamin C, as well as antibacterial, anti-inflammatory, and analgesic compounds [60, 61]. Overall, botanical, ethnobotanical, and pharmacological data are lacking on this plant species and further researches are needed to confirm its identity and better evaluate its efficacy and safety.

#### Ranula (salivary cyst)

Ranula was mentioned by 25 participants and represented 27 UR. The Tahitian name used to identify ranula was “arero ma'a,” and the French name was “double langue.”

From a medical point of view, ranula is defined as a mucus retention cyst found under the tongue. It is mainly caused by leakage of mucus from the sublingual salivary gland that is induced by a local trauma [62]. Ranula, especially plunging ranula, has a genetic basis in Māori and Polynesians, as they are significantly more affected than other ethnic groups. Ranula can appear in both adults and children [63]. Conventional therapies are mainly based on surgical treatments such as removal of the sublingual gland. Other therapies include drugs acting on the pro- and anti-inflammatory cytokines (e.g., TGF-β, TNF-α, IL-6, IL-8, IFN-γ) [64, 65].

In our work, a total of 17 unique remedies were mentioned to be used for treating ranula. Up to three ingredients were combined in these remedies. Ten plant species belonging to 10 different botanical families were reported to be used for treating ranula. The most cited plant species were *Rorippa sarmentosa* [leaf or whole plant] (8 UR), *Heliotropium arboreum* [leafbud] (5 UR), *Morinda citrifolia* [unripe and/or ripe fruit] (4 UR), *Spondias dulcis* [unripe fruit] (4 UR), and *Psidium guajava* [leafbud] (3 UR).

The most cited remedy (4 participants, 16%) was composed of *Heliotropium arboreum* leafbuds mixed with brown sugar. This remedy is crushed, put in a cloth, then pressed to get the juice, and taken orally (4 participants). Two participants use 6 leafbuds, one participant uses 3 leafbuds, and another one uses 2 leafbuds. Two participants mentioned administering the remedy for 3 days.

##### Ethnobotanical, pharmacological, and clinical data on this remedy

In Raiatea and Tahaa, *H. arboreum* leafbuds were already reported to be used for treating *arero ma'a* [3]. In Tokelau, leaves of *H. arboreum* are used for treating inflammations and swellings [66]. More generally, *H. arboreum* leaves are widely known in the Pacific for their activity against fish poisoning [67]. This activity has been attributed to the presence of rosmarinic acid [68]. Interestingly, this compound presents anti-inflammatory properties, as well as anti-edema and cytoprotective effects that could justify its use for treating ranula [69, 70]. As already mentioned in one of our previous work, *H. arboreum* contains hepatotoxic compounds (i.e., pyrrolizidine alkaloids), and their presence in the aqueous extract of *H. arboreum* leaves is still controversial [1]. Moreover, some studies have already demonstrated that children are more sensitive to these compounds than adults [71]. Therefore, the safety of *H. arboreum* leaves in children is not guaranteed, and further studies are needed to evaluate this risk.

The second most cited remedy (3 participants, 12%) was composed of the whole plant of *Rorippa sarmentosa*, the leafbuds of *Psidium guajava*, and brown sugar. This remedy is crushed, put in a cloth, and then pressed to get the juice (3 participants). This preparation is administered orally (2 participants) or applied under the tongue (1 participant). All three participants mentioned using it for 3 days. One participant mentioned mixing 3 whole plants of *Rorippa sarmentosa* with 6 leafbuds of *Psidium guajava*, and 1 teaspoon of sugar. Another one mentioned mixing 5 whole plants and 6 leafbuds. And the last one mentioned using from 2 to 3 whole plants (up to 6–8), and 2 to 4 leafbuds.

##### Ethnobotanical, pharmacological, and clinical data on this remedy

In the Society and Marquesas Islands, *R. sarmentosa* was one of the most cited plant species for treating *arero ma'a* [2–4]. In Samoa, the leaves were also used for treating inflammatory disorders in children [53]. To the best of our knowledge, *P. guajava* was not yet reported to be used in French Polynesia for ranula, but other uses related to inflammatory disorders were recorded such as otitis, urinary tract infections, respiratory disorders, and hemorrhoids [2, 3]. *R. sarmentosa* whole plant has already shown anti-inflammatory and anti-edema activities [72]. Leaves and stem of Polynesian *R. sarmentosa* (Brassicaceae) contain glucosinolates (methylsulfinylalkyl GLs, also alkenyl, arylaliphatic, and indolyl GLs) and isothiocyanates which are sulfur compounds occurring in Brassicaceae family plants and being supposed to be the active principles of this medicinal plant. Actually, isothiocyanates are known to have antibacterial properties [73]. However, pharmacological and toxicological data are scarce for this plant species. At the opposite, *P. guajava* has been widely studied. The leaves have demonstrated anti-inflammatory, analgesic, and antipyretic properties [74]. Catechin, gallic acid, and lycopene have been proposed to be responsible for the anti-inflammatory activities [75, 76]. In one clinical trial, *P. guajava* leaves significantly reduced menstrual pain in patients suffering from dysmenorrhea [77], and in another trial *P. guajava* leaves reduced knee pain in patients suffering from osteoarthritis [78]. Regarding its toxicity, *P. guajava* leaves have a low toxicity profile [79], but toxicological data in newborn babies and children are lacking. Altogether, the leaves of *P. guajava* and the whole plant of *R. sarmentosa* present ethnobotanical evidence of efficacy, as well as pharmacological data confirming their use in the treatment of inflammatory disorders. However, further studies are needed to confirm their safety when combined together.

The second (*ex-aequo*) most cited remedy (3 participants, 12%) was composed of the unripe fruit of *Spondias dulcis*. The fruits were grated, put in a cloth, then pressed, and taken orally (3 participants). Two participants mentioned using from 2 to 3 fruits. Only one reported a posology and use it for 1–3 days.

##### Ethnobotanical, pharmacological, and clinical data on this remedy:

While no previous reports of *Spondias dulcis* fruits were found in French Polynesia for treating *arero ma'a*, immature fruits are well known for treating food and fish poisoning in the area [1, 2, 4]. In Asia and the Pacific, *S. dulcis* fruits are used to treat inflammatory disorders and internal ulceration [80]. Although a few pharmacological studies have been performed on this species, other plants from *Spondias* genus have demonstrated anti-inflammatory, antipyretic, and analgesic properties [81]. Due to the lack of information regarding this species, further studies are required to evaluate its efficacy and safety.

#### Teething

Teething was mentioned by 24 participants and represented 17 UR (not all participants gave us details on used remedies). The Tahitian name for identifying teething was “niho.” This term was clearly referring to teething which could not be considered as a disease, so no additional data on the symptomatology and causes were asked.

A total of 10 unique remedies were reported to be used for treating teething including eight plant species belonging to eight botanical families. Up to three ingredients were mixed in these remedies. The most cited plant species were *Heliotropium arboreum* [leaf or leafbud] (6 UR) and *Cordyline fruticosa* [leaf] (4 UR).

The most cited remedy (5 participants, 20.8%) was composed of *Heliotropium arboreum* leafbuds mixed with brown sugar. This remedy was crushed, put in a cloth, and then pressed to get the juice (5 participants). It was either applied locally on the teeth (3 participants) or administered orally (2 participants). Two participants mentioned using 3 leafbuds but did not precise the quantity of brown sugar to be used, one participant reported using 3 leafbuds and one spoon of brown sugar, another one mentioned using 6 leafbuds and one teaspoon of brown sugar, and the last one reported using 6 leafbuds and two teaspoons of brown sugar. Only two participants detailed the posology and indicated that they use it for 3 days.

##### Ethnobotanical, pharmacological, and clinical data on this remedy

As mentioned previously, the ethnobotanical and pharmacological data available on this species confirmed its activity on inflammatory disorders and swellings, but the presence of hepatotoxic compounds calls for a better evaluation of its safety in children.

The second most cited remedy (4 participants, 16.7%) was made of *Cordyline fruticosa* leaves. The leaves are either boiled in water (2 participants) or rub in water (2 participants). All participants reported using the remedy for bathing. One reported also massage the babies/children, and another one rubs the teeth with the remedy. One participant mentioned using 10 big leaves, another one 8 leaves, and the third one 6 leaves. Two participants mentioned using this remedy for 3 days, among which one precised employing it 3 times a day.

##### Ethnobotanical, pharmacological, and clinical data on this remedy

In Tonga, the leaves of *C. fruticosa* were already reported for treating toothache [82]. They were also reported to be used for aches in Samoa [53]. Various compounds (steroidal saponins and phenols) have been isolated from *Cordyline fruticosa* leaves among which some presented TNF-α inhibitions in silico [83]. Overall, *C. fruticosa* leaves have been poorly studied and more research should be carried out to confirm its pharmacological potential.

#### Cough

Cough was mentioned by 23 participants and represented 25 UR. The Tahitian name used to identify cough was “hota,” and “toux” in French.

In total, 15 unique remedies were cited to be used for treating cough. These remedies were composed of 1 to 6 ingredients. In these remedies, 12 plant species belonging to 10 botanical families were used. The most cited plant species were *Spondias dulcis* [unripe fruit or leaf] (12 UR), *Citrus x aurantiifolia* [fruit] (3 UR), *Gardenia taitensis* [flowerbud or leaf] (3 UR), and *Heliotropium arboreum* [leaf or leafbud] (3 UR).

The most cited remedy (10 participants, 66.7%) was composed of the unripe fruits of *Spondias dulcis*. The fruits were grated, put in a cloth, and pressed to get the juice (10 participants). This juice was either used alone (7 remedies, 70%), mixed with brown sugar (2 remedies), or mixed with brown sugar and coconut water (1 remedy). Eight participants (80%) reported administering the remedy orally, one participant mentioned using it orally and applying it on the forehead and the chest, and another one reported gargling the remedy. Seven participants (70%) reported using this remedy for 3 days, among which one precised giving two teaspoons twice a day, another one precised using it 3 times a day, and a third one once a day. Another participant mentioned using the remedy until healing. To prepare the remedies, three participants used three fruits of *Spondias dulcis*, one employed five fruits and one coconut, another one employed two fruits, and a last one used one fruit. One participant mentioned that this remedy helps to clear out the lungs and throat, and another one it helps to get the mucus out.

##### Ethnobotanical, pharmacological, and clinical data on this remedy

To the best of our knowledge, no records on the use of *S. dulcis* immature fruits for cough have been found in the literature from French Polynesia. However, young shoots have been reported to be used for treating sore throat [4], and leaves have been reported to be used for oral mycosis [3]. In Tonga, the young leaves are employed for babies with difficulty swallowing [19]. In the Cook Islands, the leaves are used for treating children with thrush. In Samoa, they use the bark for the same purpose [20]. Despite the few numbers of thorough studies on the pharmacological activities of *S. dulcis*, preliminary works indicate that *S. dulcis* have antibacterial and immunomodulatory effects [84, 85]. Further researches are thus needed to confirm the efficacy of *S. dulcis* immature fruits in the treatment of cough.

The second most cited remedy (3 participants, 30%) was composed of lime juice and honey. The remedy is prepared by mixing both ingredients together, and taking it orally. No additional information was reported on this remedy.

##### Ethnobotanical, pharmacological, and clinical data on this remedy

The use of honey and lime (or lemon) juice as a cough reliever is well known all over the world [86]. *C. x aurantiifolia* fruit has been shown to possess antibacterial activity, and this activity was mainly attributed to monoterpenes such as limonene and linalool [87, 88]. It is also rich in antioxidant such as vitamin C. The second ingredient of this remedy, honey, has been widely studied for its role in cough. Its antibacterial activity has already been demonstrated, and the presence of hydrogen peroxide along with polyphenolics compounds and antimicrobial peptides such as bee defensin-1 has been proposed as bioactive compounds [89, 90]. Some clinical trials have tried to evaluate its benefits in children presenting with cough. For example, the effect of honey on nocturnal cough associated with childhood upper respiratory tract infections has been studied [91]. The authors have shown that different honey products (including one combining honey and citrus) were superior to placebo in alleviating cough. Other authors performed a meta-analysis of four randomized control trials studying the effect of honey in children (under 18) with acute cough [92]. They showed that there is moderate-certainty evidence that honey reduces cough duration to a greater extent than placebo. They also found that three days of administration is probably more effective in relieving symptoms of cough. Overall, few side effects have been reported, but *Clostridium botulinum* can contaminate honey products and thus lead to infantile botulism [93, 94]. Therefore, honey should not be given to children under 12 months old. Finally, honey should be used for a short period of time to avoid dental caries.

#### Fracture

Fracture was cited by 18 participants and represented 21 UR. Fracture was defined in Tahitian by “fati.”

A total of 14 unique remedies were reported to be used for treating *fati*. From one to six ingredients were combined in these remedies. Overall, 18 plant species belonging to 17 botanical families were reported. The most cited plant species were *Microsorum grossum* [leaf] (5 UR), *Coleus scutellarioides* [leaf] (5 UR], *Codiaeum variegatum* [leaf] (4 UR), and *Davallia solida* [rhizome] (4 UR).

The most cited remedy (5 participants, 22.2%) was made of the leaves of *Coleus scutellarioides*. The leaves were either crushed, put in iced water, then applied locally (2 participants), or boiled in water and used for bathing (2 participants), or crushed, put in a cloth, pressed, mixed with monoi, and applied locally (1 participant). Only one participant gave more details on the quantity and posology to be used and mentioned using 1 full hand of leaves and applying the remedy 1 time a day.

##### Ethnobotanical, pharmacological, and clinical data on this remedy

To the best of our knowledge, no similar uses were reported in the French Polynesian literature. In Samoa, *C. scutellarioides* leaves were reported to be used for treating sores [53]. In the Philippines, *C. scutellarioides* leaves were mentioned to be used for treating swollen muscles, fracture, and dislocation [95]. In Papua New Guinea, the leaves of *C. scutellarioides* were applied to inflammation and swellings [96]. Abietane diterpenoids (i.e., coleons O and G, lanugone K, and 6-acetylfredericone B) presenting anti-inflammatory activities (especially NF-*κ*B inhibitory activity) have been isolated from the aerial parts of *C. scutellarioides* [97]. Rosmarinic acid is also present in *C. scutellarioides*, and this compound is known to possess anti-inflammatory and antinociceptive properties [98]. Altogether, the leaves of *C. scutellarioides* present ethnobotanical evidence of efficacy, as well as pharmacological data confirming its use in the treatment of inflammatory disorders. However, the lack of toxicological data (e.g., allergy) call for more research in this area.

The second most cited remedy (3 participants, 21.4%) was composed of the leaves of *Codiaeum variegatum*. All participants mentioned boiling the leaves in water, and using it for bathing. Only one mentioned the posology and reported using it for 3 days.

##### Ethnobotanical, pharmacological, and clinical data on this remedy

In the Fiji Islands, the sap of *C. variegatum* stem is used to treat sores. In China, a leaf decoction is massaged to treat edema and drunk for its anti-inflammatory properties [99]. In Papua New Guinea, *C. variegatum* roots are massaged to treat dislocated joints [100]. Besides some poor-quality studies, no work has focused on studying the anti-inflammatory properties of *C. variegatum*. However, flavonoids present in *C. variegatum* such as apigenin, rutin, orientin, vitexin, and isovitexin have been shown to possess anti-inflammatory effects [99]. It is noteworthy that the sap of *C. variegatum* leaf can induce contact dermatitis after long term exposure [101]. Overall, *C. variegatum* leaves present poor ethnobotanical and pharmacological evidence of efficacy. Further researches are needed to better evaluate its efficacy, and precautions should be taken to ensure its safety (i.e., short time exposure only).

#### Fever

Fever was cited by 17 participants and represented 19 UR. Fever was defined in Tahitian by “fīva” which derived from the English “fever.” No additional information was obtained regarding its causes and method of diagnosis.

A total of 14 unique remedies were reported to be used for treating fever. Up to four ingredients were combined to prepare these remedies. Overall, 12 plant species belonging to 11 botanical families were reported. The most cited plant species were *Cocos nucifera* [coconut water] (6 UR) and *Thespesia populnea* [fruit] (4 UR).

The most cited remedy (4 participants, 23.5%) was coconut water alone. Three participants mentioned employing a coco of green color. Regarding the method of preparation, three participants reported using the coconut water without any preparation, while one mentioned leaving it to ferment in a bottle for 2 months. Regarding the method of administration, three participants indicated drinking the water, of which one also reported applying it locally, and another one massaging the babies/children. Another participant only mentioned applying it locally. One participant reported that this remedy helps to lower the temperature.

##### Ethnobotanical, pharmacological, and clinical data on this remedy

In French Polynesia, coconut water is widely employed in food and medicine. It is frequently used as a substitute of water and for helping diuresis [4]. In the book by Grépin and Grépin, they reported three Tahitian recipes using coconut water (in combination with other ingredients) for treating fever [17]. Coconut water is source of macronutrients such as glucose, and it also contains electrolytes such as potassium and sodium, making it a good rehydrating and moisturizing solution [102]. However, one should be aware that fever can be caused by a large number of factors (more or less severe) and that coconut water might not be enough to counteract these factors.

The second most cited remedy (3 participants, 21.4%) was composed of *Thespesia populnea* fruits. All participants reported collecting the seeds (many seeds for one participant, 20 fruits for another participant), then crushing the seeds to get the sap, and mixing it in water. One participant reported bathing the babies/children, another one mentioned applying the water on the skin, and the last one reported both methods of administration. One participant mentioned using this remedy two times a day for three days, and another one reported using it many times a day.

Of note, one participant reported that he uses a combination of *Thespesia populnea* seeds crushed in coconut water, and administers orally the remedy two times a day for three days.

##### Ethnobotanical, pharmacological, and clinical data on this remedy

In Tahiti, a similar remedy combining *T. populnea* seeds and coconut water was reported for treating fever [17]. In the Cook islands, *T. populnea* fruits were mentioned to be used for urinary tract infections and abdominal swellings, while the bark was used for ailments associated with teething in babies [20]. In Tonga, *T. populnea* bark is used to treat mouth infections in children, along with enteritis and diarrhea [82]. In Samoa, *T. populnea* bark is used to treat stomatitis [53]. In India, *T. populnea* leaf, fruit and stem bark were cited as anti-inflammatory agents [103]. Regarding its pharmacological properties, one study showed that *T. populnea* seeds possess in vivo anti-inflammatory, analgesic, and antipyretic properties [104]. Other studies focusing on *T. populnea* bark and leaves also demonstrated in vivo anti-inflammatory and analgesic effects [105, 106]. Sterol, tannins, and flavonoids (e.g., rutin, quercetin, luteolin, hesperidin) have been proposed to be responsible for these activities [104]. From a toxicological point of view, few studies are available, but one showed the presence of cytotoxic compounds (e.g., gossypol, mansonones, and populenes) in the wood and dark heartwood of *T. populnea* [107]. Moreover, allergic contact dermatitis was reported by a bowl turner using *T. populnea* wood and the sensitizing compound was identified as a new mansonone [108]. Altogether, *T. populnea* fruits present ethnobotanical evidence of efficacy in inflammatory and infectious disorders, as well as pharmacological data confirming its role in fever, pain, and inflammation. However, the presence of toxic compounds calls for more research on its toxicological impact in humans.

#### Detoxifying agents

Agents used to detoxify the body were cited by 14 participants and represented 15 UR. We classified remedies in this category when participants mentioned using it for treating *he’a* but did not mention any specific symptoms (4 participants). We also included remedies for *he’a* that were said to expel internal impurities from the body (6 participants). And finally, we included in this category, remedies that were said “pu’a roto” (literally: internal soap) remedies (2 participants).

A total of 13 unique remedies were mentioned to be used as detoxifying agents. From 2 to 17 ingredients were combined in these remedies. Overall, 22 plant species belonging to 18 botanical families were cited. The most cited plant species were *Curcuma longa* [rhizome] (10 UR), *Citrus x aurantiifolia* [fruit] (7 UR), *Syzygium malaccense* [leaf] (6 UR), *Cocos nucifera* [coconut water] (5 UR), and *Morinda citrifolia* [fruit] (3 UR).

The most cited remedy (4 participants, 28.6%) was composed of four ingredients: the leaves of *Syzygium malaccense*, the rhizome of *Curcuma longa*, lime juice, and brown sugar. All participants reported crushing the ingredients together, filtering the preparation, and then administering it orally. One participant mentioned using 21 rhizomes of *Curcuma longa*, 12 leaves of *Syzygium malaccense*, 500 g of brown sugar, 12 limes, and 5 L of water. Another participant reported mixing 8 rhizomes of *Curcuma longa*, 24 leaves of *Syzygium malaccense*, 1 kg of brown sugar, and 12 limes. A third participant mentioned using 1 rhizome of *Curcuma longa*, 30 leaves of *Syzygium malaccense*, and 12 limes. The last participant mentioned using 12 rhizomes of *Curcuma longa*, 12 leaves (6 healthy leaves and 6 leaves with gall) of *Syzygium malaccense*, 1 kg of brown sugar, and 12 limes. Two participants advised to drink the remedy until finishing the bottle, and two others advised to drink the remedy like water.

##### Ethnobotanical, pharmacological, and clinical data on this remedy

Similar remedies were already reported to be used in French Polynesia for treating *he’a*, and two ingredients (*C. longa* rhizomes and *S. malaccense* leaves) constituted the core of these recipes. In Tahiti, *C. longa* rhizomes and *S. malaccense* leaves were combined with *Pandanus* sp. roots and coconut for treating *he’a* [4]. In Moorea, *C. longa* rhizomes and *S. malaccense* leaves were mixed with coconut water and *C. aurantiifolia* fruit to treat *he’a* [22]. In Raiatea and Taha’a, *C. longa* rhizomes and *S. malaccense* leaves were also combined with *C. aurantiifolia* fruits along with *C. subcordata* bark and leaves for *he’a* [5]. In Tonga, *S. malaccense* bark was used to treat an internal illness called *kahi* that is related to *he’a* [19]. Of note, a remedy mixing *C. longa* rhizomes and *S. malaccense* leaves was reported to be used for treating hepatitis and urinary tract infections in the Cook islands [109]. Regarding their pharmacological activities, *C. longa* is widely studied for its anti-inflammatory, and curcumin was identified as the main bioactive compound [110]. *C. longa* has also demonstrated detoxifying properties by protecting liver from damage due to various toxins (e.g., aflatoxins) and by the induction of detoxifying enzymes [111, 112]. *C. longa* have been widely studied in humans for its effect on inflammatory disorders, and most of them have demonstrated positive results on inflammatory responses, pain, DNA damage, and other related symptoms [113]. *S. malaccense* leaves have also shown anti-inflammatory effect, and flavonoids (e.g., myricitrin and quercetin) have been cited as bioactive compounds [114]. Leaves of *S. malaccense* contain also 6-alkenyl or 6-alkyl-salicylic acids (anacardic and ginkgolic acids) as bioactive constituents having antimicrobial activities against Gram-positive bacteria [3]. Other activities such as antioxidant, estrogenic, and thrombolytic effect might be responsible for its action on *he’a* disorders [115–117]. Regarding their toxicity, *C. longa* is generally recognized as safe at reasonable dose, but a dose-dependent hepatotoxicity has been described. Also, side effects (e.g., diarrhea, nausea, constipation, itching) and drug interactions (e.g., anticoagulants, antibiotics) have been reported [118, 119]. A clinical trial has been carried out in 17 children (> 7 years old) with asthma, and no major adverse effects were reported (except one case of nausea) [120]. Altogether, *C. longa* rhizome and *S. malaccense* present ethnobotanical and pharmacological evidence of efficacy in inflammatory disorders and as detoxifying agents. However, side effects, drug interactions, and contraindications exist. Moreover, one should know that the high dose of sugar used in these remedies can induce health disorders if administrated too frequently and for a too long period of time.

#### Vaginal and urethral discharge

Vaginal and urethral discharge was cited by 14 participants and represented 11 UR (three participants did not mention any remedies). As already mentioned previously, the general Tahitian term “he’a” was used to refer to vaginal and urethral discharge.

A total of 7 unique remedies were reported to be used for treating vaginal and urethral discharge. From 1 to 7 ingredients were combined in these remedies. Overall, 9 plant species belonging to 8 botanical families were reported. *Curcuma longa* [rhizome] ranks first in terms of citations (9 UR), followed by *Syzygium malaccense* [leaf] (6 UR), and *Citrus x aurantiifolia* [fruit] (6 UR).

The most cited remedy (4 participants, 28.6%) was composed of four ingredients: the leaves of *Syzygium malaccense*, the rhizome of *Curcuma longa*, lime juice, and brown sugar. All participants mentioned using the same method of preparation and administration. They mix ingredients together, crush them, and filter them in a cloth to get the juice. This remedy is administered orally. Two participants mentioned that they advise to drink it like a juice every day, and two others reported administering one glass per day. Regarding the used quantity, one participant mentioned mixing 32 leaves of *Syzygium malaccense*, 1 rhizome of *Curcuma longa*, and 6 limes; another participant reported using 30 leaves of *Syzygium malaccense*, 4 rhizomes of *Curcuma longa*, and 5 limes; a third one reported using 30 leaves of *Syzygium malaccense*, 24 rhizomes of *Curcuma longa*, 24 limes, 1 kg of brown sugar, and 10.5 L of water; and the last one mentioned mixing 12 leaves of *Syzygium malaccense*, 12 rhizomes of *Curcuma longa*, 12 limes, 1 kg of brown sugar, and 1 gallon of water. Three participants reported that this remedy helps to clean up/detoxify the body, of which one added that it acts on genital cysts and menstrual bleeding. One participant mentioned that *Curcuma longa* should be used in children after 12 years old, and another participant reported that this remedy should be avoided in pregnant women. Of note, this remedy was not specific to vaginal and urethral discharge, but was also indicated for other disorders related to the *he’a* category (e.g., skin disorders and general detoxification).

##### Ethnobotanical, pharmacological, and clinical data on this remedy

The main remedy used for vaginal and urethral discharge is similar to the one used as a detoxifying agent. We invite the readers to refer to the latter section for a thorough analysis of the efficacy and toxicity of this remedy.

#### Asthma

Asthma was cited by 13 participants and represented 11 UR (two participants did not mention any remedies). Participants referred to this disorder by employing the Tahitian term “ahopau” or the French word “asthme.”

A total of 11 unique remedies were cited to be used for treating asthma. Up to 10 ingredients were combined in the remedies. Overall, 14 plant species belonging to 12 botanical families were used. The most cited plant species was *Cocos nucifera* [bark, coconut sprout, coconut water] (6 UR), followed by *Cordia subcordata* [leaf] (3 UR), and *Gardenia taitensis* [flower or flowerbud] (3 UR).

All remedies were cited only one time, so we did not discuss them here.

#### Furuncles, abscesses, and other disorders with pus exudation

Furuncles, abscesses, and other disorders with pus exudation were cited by 13 participants, and it represented 12 UR (one participant did not mention any remedies). In Tahitian, it was defined as “tui” by 8 participants.

A total of 11 unique remedies were reported to be used for treating *tui*. Up to five ingredients were used. Overall, 15 plant species belonging to 12 botanical families were reported. The most cited plant species was *Phyllanthus tenellus* [aerial part or whole plant] (3 UR), *Cocos nucifera* [coconut milk] (2 UR), and *Thespesia populnea* [fruit] (2 UR).

The most cited remedy (2 UR, 16.7%) was composed of *Phyllanthus tenellus (moemoe)* whole plant. The two participants mentioned using one full hand of plant to be boiled in water and used for bathing. Among them, one participant also added massage and water spraying. The other participant also reported that the remedy can be used until healing. One participant clearly mentioned that this plant is specific for treating *tui*.

##### Ethnobotanical, pharmacological, and clinical data on this remedy

In French Polynesia, the use of “moemoe” (*Phyllanthus* spp.) for treating furuncles or suppurative otitis has already been described. However, the vernacular name “moemoe” corresponds to different *Phyllanthus* species including *P. amarus* Schumach. & Thonn., *P. debilis* J.G.Klein ex Willd., *P. virgatus* G.Forst., and *P. urinaria* L. [2, 4, 5]. In our study, we identified this species as *P. tenellus*. Therefore, all these four species are used interchangeably by Polynesians for treating “tui.” In the Cook islands and in Samoan islands, the same vernacular name (*moemoe*) is used to identify *Phyllanthus* species and these plants are also indicated for abscesses and ear infections also called “tui” [20, 121]. Whistler suggested that “moemoe” from the Cook Islands was originally identified as *Phyllanthus virgatus* and that this name later applied to introduced herbs of the same genus such as *P. amarus*. In India, *P. virgatus* and *P. amarus* have been reported to be used for treating infectious disorders and as antiseptic [122]. *P. amarus*, *P. tenellus*, and *P. virgatus* have been widely studied for their antiviral activities against hepatitis B virus, and lignans such as niranthin, nirtetralin, hinokinin, and phyltetralin were identified as responsible for the activity [123]. A virgatusin-related compound isolated from *P. virgatus* showed antibacterial activities against Gram-positive bacteria (i.e., *Bacillus subtilis* and *Staphylococcus aureus*) [124]. In *P. amarus*, virgatusin and phyllanthin have been mentioned as responsible for the antibacterial activity [125]. In *P. urinaria*, antimicrobial activity has been associated with the presence of phyllanthin, phyltetralin, rutin, quercetin, trimethyl-3,4-dehydrochebulate and methyl brevifolincarboxylate [126]. The anti-inflammatory and anti-edematogenic effect of *P. amarus* was confirmed in a mice model of carrageenan-induced paw edema and the lignans phyltetralin, nirtetralin, niranthin were identified as bioactive compounds [127]. In *P. urinaria*, the in vitro anti-inflammatory activity was reported and eight compounds were identified as bioactives: phyllanthin, phyltetralin, trimethyl-3,4-dehydrochebulate, methylgallate, rhamnocitrin, methyl brevifolincarboxylate, quercitrin, and rutin [128]. An analgesic effect was also demonstrated in *P. tenellus* callus culture extracts [129]. Regarding their toxicological profile, *P. amarus* did not induce any side effects after 3 months of use in humans, but various toxicity effects (e.g., kidney and cardiac toxicity, contraindication with heart medications, contraindication in women seeking pregnancy) have been reported in animal models [127]. In the case of *P. tenellus*, the plant species did not induce toxicity in an acute toxicity study in mice but led to behavioral changes (i.e., agitation, spasms, depressant behavior) [130]. For *P. urinaria*, it was well tolerated by humans affected with hepatitis B, but some of them reported mild negative effects [126]. Overall, the four *Phyllanthus* species (*P. amarus*, *P. tenellus*, *P. urinaria*, and *P. virgatus*) present ethnobotanical and pharmacological evidence of efficacy in bacterial and inflammatory disorders. *Phyllanthus* lignans (e.g., phyllanthin, phyltetralin, virgatusin) seem to be the main bioactive compounds. Although rare, toxicological data suggest the overall safety of these species in adults, but the lack of study in children requires to perform more researches.

#### Otitis

Otitis was cited by 13 participants, and represented 15 UR. All participants referred to otitis by employing the French word “otite.” Some participants also referred to this disorder by employing the Tahitian terms “tari’a ma’i” (4 participants) or “tui” (2 participants).

A total of 13 unique remedies were reported to be used for treating otitis. Up to four ingredients were used in these remedies. Overall, 11 plant species belonging to 10 botanical families were reported. *Phyllanthus tenellus* [leaf or whole plant] was the most cited plant species (3 UR), followed by *Cocos nucifera* [coconut milk or coconut water] (2 UR), *Morinda citrifolia* [fruit] (2 UR), and *Zingiber officinale* [rhizome] (2 UR). Besides the use of plants, monoi (a Polynesian cosmetic product made from coconut oil and specific flowers, especially from *Gardenia taitensis*) was reported by seven participants for treating otitis. It was either used in combination with plants (4 UR) or mixed with a centipede (2 participants), or used alone (1 participant).

The most cited remedy (2 UR) was composed of *Phyllanthus tenellus* whole plant and monoi. The plant was crushed, put in a cloth, pressed, then mixed with monoi, and applied on the affected ear. One participant reported using one full hand of plant.

##### Ethnobotanical, pharmacological, and clinical data on this remedy

The main remedy used for otitis is similar to the one used for furuncles, abscesses, and other disorders with pus exudation. We invite the readers to refer to the latter section for a thorough analysis of the efficacy and toxicity assessment of this remedy.

The second (*ex-aequo*) most cited remedy (2 UR) was composed of monoi and a centipede. The centipede is placed alive in a bottle full of monoi, the mixture is kept for some weeks, and then, the liquid can be applied on the ear. One participant applies 1 drop 2 times a day for 2–4 days. The remedy was said to stop exudation and earache.

##### Ethnobotanical, pharmacological, and clinical data on this remedy

To the best of our knowledge, no remedies using centipede have been recorded before in French Polynesia. Peptides isolated from centipede toxins or from the whole centipede have been shown to possess analgesic and antimicrobial activities that might explain their use in medicine for earache [131]. However, the lack of toxicological data available in children and the presence of toxins in the centipede incite to avoid using this remedy in children.

#### Lower abdominal disorders

Lower abdominal disorders were mentioned by 12 participants, and it represented 13 UR. All participants refer to this disorder by using the Tahitian term “tia” (meaning the bottom of the belly).

A total of 13 unique remedies were mentioned to be used for treating lower abdominal disorders. Up to four ingredients were combined in these remedies. Overall, 11 plant species belonging to 11 botanical families were reported. The most cited plant species was *Saccharum officinarum* [stem] (6 UR), followed by *Cocos nucifera* [coconut water] (4 UR), *Citrus x aurantiifolia* [fruit] (3 UR), and *Cordyline fruticosa* [leaf, or leafbud] (3 UR).

All remedies mentioned by participants were mentioned only one time each. Of note, one participant reported administering sugarcane juice, and another one mentioned administering coconut water. The latter one also reported that coconut water help to clean the urine. Overall, the remedies used for treating lower abdominal disorders were mainly based on the use of beverages made from coconut water, sugarcane, or lime juice. This is in accordance with the symptoms cited that are mainly related to urinary infections, and the fact that these types of infections require an increase in diuresis and therefore drinking a lot of water. Moreover, coconut water, sugarcane, and lime juice are also used as food and so are generally recognized as safe.

#### Skin disorders (associated with *he'a*)

Skin disorders (associated with *he'a*) were mentioned by 12 participants and represented 11 UR (one participant did not mention any remedies). All participants referred to this disorder by employing the Tahitian term “he'a,” and by precising its skin localization.

A total of 11 unique remedies were mentioned to be used for treating skin disorders (associated with *he'a*). Up to seven ingredients were used in these remedies. Overall, 13 plant species belonging to 11 botanical families were reported. The most cited plant species was *Cordia subcordata* [leaf] (5 UR) followed by *Saccharum officinarum* [stem] (5 UR), *Citrus x aurantiifolia* [fruit] (3 UR), and *Ficus tinctoria* [fig] (3 UR).

Although none of the remedies were cited more than one time, we can note that the leaves of *Cordia subcordata* and the sap of *Ficus tinctoria* figs were associated in three different remedies. All remedies had the same method of preparation which consisted of pressing *F. tinctoria* figs above a *C. subcordata* leaf, so the latex of *F. tinctoria* falls into the leaves of *C. subcordata*. The remedy was described to be associated with different plant species (e.g., *Gardenia taitensis*, *Heliotropium arboreum*, *Saccharum officinarum*, *Thespesia populnea*).

##### Ethnobotanical, pharmacological, and clinical data on this remedy

Interestingly, *F. tinctoria* and *C. subcordata* are widely known in French Polynesia for dying textiles. To prepare the dying solution, the same preparation mixing the juice of *F. tinctoria* figs and the leaves of *C. subcordata* is employed. This might indicate that the remedy used in medicine originated from the dying preparation. In the literature, *C. subcordata* leaves were already reported to be used for treating infected wounds in Tahiti [4, 14]. In the Marquesan Islands, *C. subcordata* fruits were reported to treat itching, skin allergy, and wounds [132]. In Raiatea and Tahaa, the latex from *F. tinctoria* was mentioned to be used for acne and furuncles [5]. Although very few pharmacological studies have been performed on these two species, latex from other *Ficus* species has already demonstrated wound healing activity [133]. On the other hand, studies have shown the allergen potential of latex from some *Ficus* species [134]. Therefore, further researches are needed on this remedy to confirm its efficacy and its safety in children.

#### Umbilical cord care

Umbilical cord care was mentioned by 12 participants, and it represented a total of 8 UR (four participants did not cite any remedies). Of all participants, 10 (83.3%) referred to this treatment by using the Tahitian term “pito” (meaning umbilicus, navel), and the two others referred to this treatment by using the French term “ombilic” (umbilicus) or “nombril” (navel). Four participants reported that this treatment is associated with umbilical cord care in newborn babies.

A total of 7 unique remedies were mentioned to be used for umbilical cord care. Up to four ingredients were combined in these remedies. Overall, 7 plant species belonging to 7 botanical families were reported. The most cited plant species was *Persicaria glabra* [aerial part, or leafbud, or stem, or stembud] (4 UR), followed by *Saccharum officinarum* [stem] (4 UR), and *Gardenia taitensis* [flowerbud, leaf] (3 UR).

Only one remedy was cited more than one time. This remedy was cited by two participants and was composed of *Persicaria glabra* stem, *Gardenia taitensis* flowerbud, and sugarcane. All participants reported crushing the plant species, mixing them together, and administering the remedy orally for 3 days. One precised using 3 tablespoons a day. The other also mentioned applying the remedy on the navel. Regarding the posology, one participant reported using 2 stems of *Persicaria glabra*, 2 flowerbuds of *Gardenia taitensis*, and 1 internode of *Saccharum officinarum* stem.

##### Ethnobotanical, pharmacological, and clinical data on this remedy

No records on the use of *G. taitensis* flowerbuds and *P. glabra* stem either prepared separately or mixed together were found in the literature from Polynesia for treating umbilical cord care. Also, records of medicinal uses for *P. glabra* stem are limited in the Polynesian literature. However, Pétard mentioned a remedy using *P. glabra* leaves for cutaneous mycosis [4]. Regarding *G. taitensis*, flowerbuds have already been cited to be used for treating skin disorders such as sores, infected wounds, or abscesses in the Society and the Marquesas Islands [2, 5, 14]. In Tonga, *G. taitensis* leaves were applied to sores on the skin [19]. In Samoa, the crushed leaves or flowers were applied on skin inflammation [53]. In Ayurvedic medicine, *P. glabra* stem has been used for arthritic and inflammatory disorders. Anti-inflammatory activities of *P. glabra* stem have already been demonstrated in different in vivo models including the carrageenan-induced paw edema model [135]. In the same study, *P. glabra* stem was administrated for 30 days to rats and no significant toxicity events were noticed. Also, the antibacterial effect of *P. glabra* leaves was demonstrated in vitro, and the bioactive compounds were identified as quercetin and isorhamnetin derivatives [136]. Of note, a new butenolide cinnamate with anti-mycobacterial and antiviral activities was also identified in *P. glabra* aerial parts [137]. To the best of our knowledge, no pharmacological studies have been published on *G. taitensis*. However, over 150 volatile compounds have been isolated from the flowerbuds including linalool and isoeugenol (compounds with antimicrobial activities), along with salicylate derivatives (compounds with anti-inflammatory and analgesic activities) [138]. Altogether, *G. taitensis* present ethnobotanical and phytochemical evidence of efficacy in inflammatory and infectious skin disorders, while *P. glabra* present pharmacological evidence of efficacy in inflammatory and anti-infectious disorders. Although *P. glabra* seems to be safe for an external use, the presence of salicylates in *G. taitensis* might induce some toxicity in children under certain conditions. For example, the Reye syndrome is a fatal disease that occurs in children using salicylate and having viral infections. Although rare and unlikely to happen for an external use, it is worth mentioning such toxicity. Also, further studies are needed to better understand the effect of the combination of both plants on wound healing and skin infections.

#### Chickenpox

Chickenpox was mentioned by 11 participants and represented 13 UR. It was also the most frequently cited disorder from the infectious disease’s category. All participants referred to this disorder by using the French term “varicelle” (meaning chickenpox), and five participants also used the Tahitian term “'ōniho” (meaning chickenpox).

A total of 5 unique remedies were reported to be used for treating chickenpox. All remedies were composed of only one ingredient. Overall, five plant species belonging to four botanical families were reported. The most cited plant species was *Calophyllum inophyllum* [leaf] (7 UR), followed by *Annona muricata* [leaf] (2 UR), and *Hibiscus tiliaceus* [flower] (2 UR).

The most cited remedy (7 UR) was composed of the leaves of *Calophyllum inophyllum*. The plant is either rubbed in water (4 UR), crushed (2 UR), or boiled in water (1 UR). All participants reported using the remedy for bathing the children. Of the four participants mentioning the quantity of leaves used, two reported using 30 leaves, one reported using 20 leaves, and another one mentioned 5 leaves. One participant reported that this remedy helps to cicatrize, and a second one mentioned that it softens the skin and helps heal the wound.

##### Ethnobotanical, pharmacological, and clinical data on this remedy

*Calophyllum inophyllum* leaves are widely used in Polynesia for treating skin ailments. In the Society Islands, the leaves are boiled and prepared for bathing patients presenting herpes, eczema, itches, or intertrigo [4]. In the Marquesan islands, the leaves are used to treat itches, skin allergy, burns, mild wounds, and scabies [2]. In Raiatea and Tahaa, the leaves are used as a bath for treating cutaneous symptoms and mycosis [3]. In Samoa, the leaves are used to bathe patients with skin rash or infections [121]. In the Cook Islands, the leaves crushed in seawater are employed for bathing patients with skin sores and rashes [20]. Different class of phytochemicals were reported to be found in *C. inophyllum* leaves, such as triterpenoids, steroids, flavonoids, coumarins, xanthones, fatty acids, esters, alkenes, ethers, and alicyclic compounds. These secondary metabolites from *C. inophyllum* are known for their bioactivity, such as antiviral, anti-inflammatory, antimicrobial, analgesic, and as photo-protective agents [139–141]. The anti-inflammatory properties of *C. inophyllum* leaves were already demonstrated on lipopolysaccharide (LPS)-induced RAW 264.7 cells, and triterpenoids compounds were identified as responsible for the activity with 27-[(E)-p-coumaroyloxy]canophyllic acid being the most active one [142]. Wound healing and antibacterial activities were also described for *C. inophyllum* oil from different Polynesians origins [143]. Also, calophyllolide from *C. inophyllum* seeds was identified as responsible for the wound healing and anti-inflammatory activities [144]. It was reported that calophyllolide is also present in *C. inophyllum* leaves but at a lower concentration than in seeds [145]. It was also demonstrated that *C. inophyllum* oil is safe to apply topically at a therapeutic dose [143]. However, allergic contact dermatitis was already diagnosed from a patient applying tamanu oil samples [146]. Altogether, *C. inophyllum* leaves present ethnobotanical and pharmacological evidence of efficacy in infectious and inflammatory skin disorders. As the decoction of *C. inophyllum* leaves in water is likely to contain less concentrated compounds than in *C. inophyllum* oil, we can hypothesize that the decoction of *C. inophyllum* leaves use as a bath is safe at reasonable doses, although allergic reactions may occur.

#### Effects of reduced temperature

This disorder was mentioned by 11 participants, and it represented 9 UR. (two participants did not cite any remedies.) Nine participants (81.8%) referred to this disorder by using the Tahitian term “puta to'eto'e” (literally: being penetrated by the cold), of which one specified that this disorder belongs to an “ira” category. Eight participants referred to this disorder by using the French term “attraper froid” (literally: catching cold).

A total of 7 unique remedies were used for treating this disorder. Up to three ingredients were mixed in this remedy. Overall, nine plant species belonging to eight botanical families were reported. The most cited plant species was *Coffea arabica* [leaf] (2 UR), followed by *Ocimum basilicum* [aerial part] (2 UR).

The most cited remedy (2 UR) was composed of the coffee tree leaves. This remedy is boiled in water and either used as a steam bath or for bathing. No quantity of plant materials to be used and no posology was specified by the participants. One participant mentioned that this remedy can help eliminate cold sweat.

##### Ethnobotanical, pharmacological, and clinical data on this remedy

To the best of our knowledge, no reports were found on the use of *C. arabica* leaves for similar disorders (cold, fever, shivers) in Polynesia. In the Marquesas Islands, *C. arabica* leaves were mentioned to be used for treating diabetes and gout [2]. In Haiti, the decoction of *C. arabica* leaves taken orally or applied locally was reported to be used for treating headache. In Mexico, the leaves prepared into a poultice are used to treat fever. In Nicaragua, *C. arabica* leaves are used externally for headache [147]. Not only, caffeine is present in *C. arabica* seeds but it is also present in leaves [148]. Caffeine has been widely studied for its effect on migraine, and is currently associated with paracetamol or aspirin for this therapeutic use in over-the-counter medicines. Caffeine is also known as a central nervous system stimulant and thus can help patients recover from benign medical conditions. Mangiferin was also detected in *C. arabica* leaves, and this compound possesses anti-inflammatory properties [149]. The EFSA (European Food Safety Authority) evaluated the use of *C. arabica* leaves infusion as a traditional food, and they did not report any safety risks for the population, except one case of allergic reaction [148]. Overall, *C. arabica* leaves present ethnobotanical and pharmacological evidence of efficacy in migraine and inflammatory conditions. Although allergic reactions can occur, the use of *C. arabica* leaves as a bath or steam bath seem safe at reasonable doses, but data in children are lacking.

The second (*ex-aequo*) most cited remedy (2 UR) was composed of the aerial part from basil. One full hand of aerial parts is collected, then boiled in water, and used as a steam bath. One participant mentioned using it one time a day for three days.

##### Ethnobotanical, pharmacological, and clinical data on this remedy

In the Marquesans islands, *O. basilicum* leaves were mentioned to be used for cold, influenza, and sore throat [2]. In the Cook islands, *O. basilicum* leaves boiled in water are used orally to treat urinary tract infections [20]. In Tahiti and in the Cook islands, a bath or massage with a solution of *O. basilicum* leaves was also used to chase away ghosts who possessed someone [14, 20]. In Guatemala, a decoction of leaves is used for coughs and phlegm [150]. In Mexico, aerial parts of *O. basilicum* are used to treat fever [151]. In Colombia, *O. basilicum* leaves are used for bronchial infections and common cold [152]. Analgesic activity of *O. basilicum* was demonstrated in mice using different models (acetic acid-induced writhing test, hot plate test, tail immersion technique). Eugenol and linalool were described as responsible for this activity. Anti-inflammatory effect was also demonstrated in several in vitro and in vivo models, and this activity was attributed to compounds such as α-bergamotene, α-cadinol, estragole, eugenol, linoleic acid, methyl cinnamate, and methyl eugenol. Antibacterial activity of *O. basilicum* essential oil was demonstrated in vitro, and α-terpineol, 1,8-cineole, estragole, eugenol, and linalool were identified as bioactive compounds. A broad spectrum of antiviral activities was also shown using the crude aqueous extract and ethanolic extract of *O. basilicum*. Apigenin, eugenol, linalool, and especially ursolic acid were identified as responsible for this activity [153]. The presence of linalool and eugenol was also shown to be responsible for the relaxant and bronchodilatory effects of *O. basilicum*. A safety analysis showed that *O. basilicum* is safe for humans at recommended doses [154]. However, the presence of 1,8-cineole (eucalyptol) that is known to induce convulsions and blanks in newborn babies makes the use of *O. basilicum* steam bath inappropriate to children under 3 years old [155].

## Conclusion

In this article, we present the results of an ethnobotanical survey that was designed to document the remedies used for children in the Society Islands. We then performed a thorough bibliographic search to assess their efficacy and safety.

Out of the 29 plant uses evaluated, 8 presented ethnobotanical evidence of efficacy only, 1 presented pharmacological evidence of efficacy only, 18 presented ethnobotanical and pharmacological evidence of efficacy, and 2 could not be evaluated due to the lack of studies. Regarding their toxicity, 3 had a good safety profile, 8 presented a potential risk of toxicity, one presented a high risk of toxicity, and 17 could not be evaluated due to the lack of studies. *Microsorum grossum* (metuapua’a) present clear past clinical evidence of toxicity in children, and its use should be avoided for children, and even for adults in the absence of safety data. Also, brown sugar is frequently used to improve the taste of the remedy but this can induce health disorders if administrated too frequently and for a too long period of time. It is worth mentioning that this efficacy and risk assessment was mainly based on the results of preclinical studies (in vitro and animal studies) and thus might not be the reflection of human responses.

In conclusion, more researches (especially clinical studies) should be performed on the plants mentioned in this study as most of them have been poorly studied in children. Also, dedicated programs should be performed in French Polynesia to communicate about the benefits and risks of using traditional remedies in children.

### Supplementary Information

**Additional file 1: Table S1.** Informant Consensus Factor for health conditions cited more than 10 times.

**Additional file 2: Table S2.** Fidelity Level for plant species cited more than five times.

**Additional file 3: Table S3.** Summary of the efficacy and toxicity assessment of the most cited plants associated with their main uses (based on a bibliographic review).

**Additional file 4: Table S4.** Overview of the number of ingredients per remedy for the most cited children illnesses.

## Data Availability

The datasets used and/or analyzed during the current study are available from the corresponding author on reasonable request.

## Abbreviations

5-HT: 5-Hydroxytryptamine receptors
ABS: Access benefit sharing
CITES: Convention on International Trade in Endangered Species of wild fauna and flora
DIREN: Direction of Environment from French Polynesia
FL: Fidelity level
GABA: Gamma-aminobutyric acid
GLs: Glucosinolates
ICD: International Classification of Diseases
ICF: Informant Consensus Factor
IFN: Interferon
IL: Interleukin
Ind.: Indigenous
IUCN: International Union for Conservation of Nature
LPS: Lipopolysaccharide
Mod.: Modern introduction
ND: Not documented
NF-*κ*B: Nuclear factor-kappa B
NI: Not Identified
Pol.: Polynesian introduction
TGF: Transforming growth factor
TNF: Tumor necrosis factor
UR: Use-reports

## Footnotes

1 Here, the term “*Hibiscus rosa-sinensis*” is used to designate the complex *Hibiscus rosa-sinensis* that have recently been discovered as a species complex including various endemic Pacific specie*s,* cultivars and hybrids [47]*.*
