# Supplementary material for: Traditional medical practices for children in five islands from the Society archipelago (French Polynesia)
Source: J Ethnobiol Ethnomed. 2023 Oct 18;19:44. doi: 10.1186/s13002-023-00617-0 (PMC10585756; doi:10.1186/s13002-023-00617-0)
Supplement: Supplementary file 1 — Additional file 1: Table S1. Informant Consensus Factor for health conditions cited more than 10 times. [file 13002_2023_617_MOESM1_ESM.docx]

**Traditional medical practices for childhood diseases in five islands from the Society archipelago (French Polynesia)**

CHASSAGNE François^1,2*^, BUTAUD Jean-François^3^, HO Raimana^4^, CONTE Eric^2^, HNAWIA Édouard^5^, RAHARIVELOMANANA Phila^4^

^1^ UMR 152 PharmaDev, Université Paul Sabatier, Institut de Recherche pour le Développement (IRD), Toulouse, France

^2^ Maison des Sciences de l’Homme du Pacifique (UAR 2503), Université de la Polynésie Française / Centre National de la Recherche Scientifique, Tahiti, Polynésie Française

^3^ Correspondant du Muséum National d’Histoire Naturelle (PatriNat), Paris & Consultant en foresterie et botanique polynésienne, Tahiti, Polynésie française

^4^ UMR 214 EIO, Université de Polynésie Française, IFREMER, ILM, IRD, BP 6570, F-98702 Faaa, Tahiti, Polynésie française.

^5^ UMR 152 PharmaDev, Institut de Recherche pour le Développement (IRD), Noumea, New Caledonia.

*Corresponding author :

François Chassagne

Université Paul Sabatier

Faculté de Pharmacie

35 Chemin des Maraîchers

31062 Cedex 09

Toulouse

FRANCE

[francois.chassagne@ird.fr](mailto:francois.chassagne@ird.fr)

**Additional file 1: Table S1**: Informant Consensus Factor for health conditions cited more than 10 times

| **Diseases** | **Number of UR** | **Number of plant species used** | **Informant Consensus Factor** |
| --- | --- | --- | --- |
| Restlessness, irritability, jerk | 158 | 23 | 0,86 |
| Tonsilitis, sore throat | 11 | 3 | 0,80 |
| he'a | 124 | 28 | 0,78 |
| Chickenpox | 12 | 4 | 0,73 |
| Vaginal and urethral discharge | 24 | 8 | 0,70 |
| Ranula (salivary cyst) | 29 | 10 | 0,68 |
| Cough | 31 | 12 | 0,63 |
| Teething | 20 | 8 | 0,63 |
| Lower abdominal disorders | 28 | 11 | 0,63 |
| Covid-19 | 14 | 6 | 0,62 |
| Sinusitis | 50 | 20 | 0,61 |
| Skin disorders (associated with he'a) | 29 | 13 | 0,57 |
| Umbilical cord care | 15 | 7 | 0,57 |
| Detoxifying agent | 54 | 25 | 0,55 |
| Fracture | 37 | 18 | 0,53 |
| Fever | 19 | 11 | 0,44 |
| Asthma | 24 | 14 | 0,43 |
| Otitis | 16 | 11 | 0,33 |
| Sprain | 11 | 8 | 0,30 |
| Furuncles, abscess and other disorders with pus exudation | 19 | 15 | 0,22 |
| Unspecified effects of reduced temperature | 11 | 9 | 0,20 |

Legend: UR = Use-Reports
