# Supplementary material for: Traditional medical practices for children in five islands from the Society archipelago (French Polynesia)
Source: J Ethnobiol Ethnomed. 2023 Oct 18;19:44. doi: 10.1186/s13002-023-00617-0 (PMC10585756; doi:10.1186/s13002-023-00617-0)
Supplement: Supplementary file 2 — Additional file 2: Table S2. Fidelity Level for plant species cited more than five times. [file 13002_2023_617_MOESM2_ESM.docx]

**Traditional medical practices for childhood diseases in five islands from the Society archipelago (French Polynesia)**

CHASSAGNE François^1,2*^, BUTAUD Jean-François^3^, HO Raimana^4^, CONTE Eric^2^, HNAWIA Édouard^5^, RAHARIVELOMANANA Phila^4^

^1^ UMR 152 PharmaDev, Université Paul Sabatier, Institut de Recherche pour le Développement (IRD), Toulouse, France

^2^ Maison des Sciences de l’Homme du Pacifique (UAR 2503), Université de la Polynésie Française / Centre National de la Recherche Scientifique, Tahiti, Polynésie Française

^3^ Correspondant du Muséum National d’Histoire Naturelle (PatriNat), Paris & Consultant en foresterie et botanique polynésienne, Tahiti, Polynésie française

^4^ UMR 214 EIO, Université de Polynésie Française, IFREMER, ILM, IRD, BP 6570, F-98702 Faaa, Tahiti, Polynésie française.

^5^ UMR 152 PharmaDev, Institut de Recherche pour le Développement (IRD), Noumea, New Caledonia.

*Corresponding author :

François Chassagne

Université Paul Sabatier

Faculté de Pharmacie

35 Chemin des Maraîchers

31062 Cedex 09

Toulouse

FRANCE

[francois.chassagne@ird.fr](mailto:francois.chassagne@ird.fr)

**Additional file 2: Table S2**: Fidelity Level for plant species cited more than five times

*The plant called “moemoe” in Tahitian includes different species such as *Phyllanthus amarus*, *P. debilis*, *P. tenellus*, *P. virgatus*, and *P. urinaria*. In our study, we only collected *P. tenellus*, so we kept this name in the table.

| **Plant species** | **Diseases** | **Number of participants citing the plant species for a given disease** | **Number of participants citing the plant species for all types of diseases** | **Fidelity Level** |
| --- | --- | --- | --- | --- |
| *Annona muricata* | Restlessness, irritability, jerk | 33 | 33 | 100,0 |
| *Rosa sp.* | Sinusitis | 7 | 7 | 100,0 |
| *Hibiscus rosa-sinensis* ‘Carnation’ | Restlessness, irritability, jerk | 29 | 33 | 87,9 |
| *Syzygium malaccense* | *he'a* | 15 | 18 | 83,3 |
| *Curcuma longa* | *he'a* | 24 | 29 | 82,8 |
| *Gardenia taitensis* | Restlessness, irritability, jerk | 28 | 40 | 70,0 |
| *Cyperus javanicus* | Restlessness, irritability, jerk | 4 | 6 | 66,7 |
| *Coleus scutellarioides* | Fracture | 5 | 8 | 62,5 |
| *Ocimum basilicum* | Restlessness, irritability, jerk | 3 | 5 | 60,0 |
| *Zingiber officinale* | Covid-19 | 3 | 5 | 60,0 |
| *Calophyllum inophyllum* | Chickenpox | 7 | 12 | 58,3 |
| *Saccharum officinarum* | Restlessness, irritability, jerk | 19 | 33 | 57,6 |
| *Rorippa sarmentosa* | Ranula | 8 | 15 | 53,3 |
| *Spondias dulcis* | Cough | 12 | 23 | 52,2 |
| *Microsorum grossum* | Restlessness, irritability, jerk | 11 | 24 | 45,8 |
| *Persicaria glabra* | Umbilical cord care | 4 | 9 | 44,4 |
| *Ficus tinctoria* | Skin disorders (he'a) | 3 | 7 | 42,9 |
| *Heliotropium arboreum* | Teething | 6 | 14 | 42,9 |
| *Phyllanthus tenellus** | Furuncles, abscess and others disorders with pus exudation | 3 | 7 | 42,9 |
| *Phyllanthus tenellus** | Otitis | 3 | 7 | 42,9 |
| *Davallia solida* | Fracture | 4 | 10 | 40,0 |
| *Leucas decemdentata* | Sinusitis | 3 | 8 | 37,5 |
| *Adenostemma viscosum* | Detoxifying agent (he'a) | 2 | 6 | 33,3 |
| *Syzygium malaccense* | Detoxifying agent (he'a) | 6 | 18 | 33,3 |
| *Curcuma longa* | Detoxifying agent (he'a) | 9 | 29 | 31,0 |
| *Psidium guajava* | Restlessness, irritability, jerk | 4 | 13 | 30,8 |
| *Aleurites moluccanus* | Lower abdominal disorders | 2 | 7 | 28,6 |
| *Hibiscus tiliaceus* | Chickenpox | 2 | 7 | 28,6 |
| *Morinda citrifolia* | Ranula | 4 | 14 | 28,6 |
| *Torenia crustacea* | Lower abdominal disorders | 2 | 7 | 28,6 |
| *Thespesia populnea* | fever | 4 | 15 | 26,7 |
| *Artocarpus altilis* | Fracture | 2 | 8 | 25,0 |
| *Cordyline fruticosa* | Restlessness, irritability, jerk | 6 | 25 | 24,0 |
| *Cordia subcordata* | Skin disorders (he'a) | 5 | 21 | 23,8 |
| *Cocos nucifera* | Sinusitis | 11 | 47 | 23,4 |
| *Citrus x aurantiifolia* | Detoxifying agent (he'a) | 7 | 30 | 23,3 |
