# Supplementary material for: Traditional medical practices for children in five islands from the Society archipelago (French Polynesia)
Source: J Ethnobiol Ethnomed. 2023 Oct 18;19:44. doi: 10.1186/s13002-023-00617-0 (PMC10585756; doi:10.1186/s13002-023-00617-0)
Supplement: Supplementary file 3 — Additional file 3: Table S3. Summary of the efficacy and toxicity assessment of the most cited plants associated with their main uses (based on a bibliographic review). [file 13002_2023_617_MOESM3_ESM.docx]

**Traditional medical practices for childhood diseases in five islands from the Society archipelago (French Polynesia)**

CHASSAGNE François^1,2*^, BUTAUD Jean-François^3^, HO Raimana^4^, CONTE Eric^2^, HNAWIA Édouard^5^, RAHARIVELOMANANA Phila^4^

^1^ UMR 152 PharmaDev, Université Paul Sabatier, Institut de Recherche pour le Développement (IRD), Toulouse, France

^2^ Maison des Sciences de l’Homme du Pacifique (UAR 2503), Université de la Polynésie Française / Centre National de la Recherche Scientifique, Tahiti, Polynésie Française

^3^ Correspondant du Muséum National d’Histoire Naturelle (PatriNat), Paris & Consultant en foresterie et botanique polynésienne, Tahiti, Polynésie française

^4^ UMR 214 EIO, Université de Polynésie Française, IFREMER, ILM, IRD, BP 6570, F-98702 Faaa, Tahiti, Polynésie française.

^5^ UMR 152 PharmaDev, Institut de Recherche pour le Développement (IRD), Noumea, New Caledonia.

*Corresponding author :

François Chassagne

Université Paul Sabatier

Faculté de Pharmacie

35 Chemin des Maraîchers

31062 Cedex 09

Toulouse

FRANCE

[francois.chassagne@ird.fr](mailto:francois.chassagne@ird.fr)

**Additional file 3: Table S3**: Summary of the efficacy and toxicity assessment of the most cited plants associated to their main uses (based on a bibliographic review)

*Note 1 : Although some remedies are a mixture of two or more ingredients, we decided to provide an analysis of each plant separately for more accuracy.*

*Note 2 : Before reading this table, one should know that allergic reactions and adverse effects can occur in any types of medicines, including all plants and ingredients presented here. Their efficacy and safety can also varies depending on some potential microbial contamination, adulteration, presence of toxins or heavy metals, phytochemical variation, and the dosage used. Finally, readers should be aware that specific types of populations (infant, children, pregnant women, breastfeeding women, elderly) are more vulnerable than healthy adults. In case of doubt or if symptoms persist, a healthcare professional should be contacted for further advice.*

| **Plants species (part of plant used)** | **Main ethnobotanical use reported in our survey** | **Efficacy** (similar ethnobotanical uses in other locations, relevant pharmacological activities, bioactive compounds, clinical trials) | | | | **Toxicity** (red : high risk of toxicity; yellow: potential risk of toxicity; green: no or low risk of toxicity; grey: risk unknown) |
| --- | --- | --- | --- | --- | --- | --- |
|  |  | **Ethno-botanical uses** | **Pharmaco-logical activities** | **Bioactive compounds** | **Clinical trials** |  |
| *Annona muricata* (leaf) | *Ira* (restlessness, irritability, to wake with a start, and convulsion) | X  (sedative) | X  (anxiolytic) | X (isoquinoline-type alkaloids) |  | Neurotoxicity due to acetogenins Lack of data in children |
| *Calophyllum inophyllum* (leaf) | Chickenpox | X (burns, eczema, scabies, itches, skin rashes, skin sores) | X (anti-inflammatory, wound healing) | X (calophyllolide, triterpenoids) |  | Risk of contact allergy Lack of data in children |
| Centipede in monoi | Otitis |  | X (analgesic, antimicrobial) | X (peptides) |  | Presence of toxins in the centipede  Lack of data in children |
| *Cocos nucifera* (coconut water) | Fever | X (water substitute) |  | X (carbohydrates, electrolytes) |  | Precautions: use as a supplement (rehydration) to a medical follow-up |
| *Codiaeum variegatum* (leaf) | Fracture | X (extravasated blood, inflammation) | X (anti-inflammatory) | X (apigenin, rutin, orientin, vitexin, and isovitexin) |  | Risk of contact dermatitis after long time exposure. Lack of data in children |
| *Coffea arabica* (leaf) | *Puta to'e to'e* (cold, shivers, headache) | X (fever, headache) | X (anti-inflammatory, anti-migraine, CNS stimulant) | X (caffeine, mangiferin) |  | Risk of contact allergy. Lack of data in children |
| *Coleus scutellarioides (leaf)* | *Fracture* | X (fracture, inflammation, swellings) | X (anti-inflammatory, anti-nociceptive) | X (abietane diterpenoids, rosmarinic acid) |  | Lack of data in children |
| *Cordia subcordata* (leaf) | Skin disorders | X (itching, skin allergy, wounds) |  |  |  | Lack of data in children |
| *Cordyline fruticosa* (leaf) | *Ira* (restlessness, irritability, to wake with a start, and convulsion) | X (fever, aches) |  |  |  | Lack of data in children |
| *Cordyline fruticosa* (leaf) | Teething | X (tootache, aches) |  |  |  | Lack of data in children |
| *Curcuma longa* (rhizome) | *He'a* (detoxifying agent, skin disorders and disorders of the genitourinary system) | X (*he'a*, inflammation, urinary tract infections) | X (anti-inflammatory, detoxifying properties) | X (curcumin) | X (chronic arsenic exposure, chronic prostatitis, pruritis, psoriasis, vitiligo) | Safe in children > 7 years old at 30 mg/kg/day.  Side-effects: constipation, diarrhea, itching, nausea Drug interactions: antibiotics, anticoagulants Precautions: sugar overload in the *he'a* remedy |
| *Ficus tinctoria* (fig) | Skin disorders | X (acne, furuncles) |  |  |  | Potential risk of contact allergy. Lack of data in children |
| *Gardenia taitensis* (flowerbud) | Umbilical cord care | X (infected wound, skin inflammation, skin sores) | X (analgesic, anti-inflammatory, antimicrobial) | X (eugenol, linalool, salicylates derivatives) |  | Risk of Reye's syndrome. Lack of data in children |
| *Gardenia taitensis* (leaf) | *Ira* (restlessness, irritability, to wake with a start, and convulsion) | X  (*ira*) |  |  |  | Risk of Reye's syndrome if presence of salicylates derivatives is confirmed in leaves. Lack of data in children |
| *Heliotropium arboreum* (leafbud) | Ranula  (salivary cyst) | X (inflammation, swellings) | X (anti-inflammatory, anti-edema) | X (rosmarinic acid) |  | Hepatotoxicity due to pyrrolizidine alkaloids. Lack of data in children |
| *Heliotropium arboreum* (leafbud) | Teething | X (inflammation, swellings) | X (anti-inflammatory, anti-edema) | X (rosmarinic acid) |  | Hepatotoxicity due to pyrrolizidine alkaloids. Lack of data in children |
| *Hibiscus rosa-sinensis* (leaf) | *Ira* (restlessness, irritability, to wake with a start, and convulsion) | X (sedative, child sleepless-ness) |  |  |  | Hepato-renal toxicity at a dose of 800 mg/kg for 14 days in mice. Lack of data in children |
| Honey and lime juice | Cough | X (cough reliever) | X (antibacterial, antioxidant) | X (limonene, linalool, bee-defensin, hydrogen peroxide) | X (cough) | Contraindication: < 12 months old (risk of botulism) Precautions: sugar overload |
| *Microsorum grossum* (leaf) | *Ira* (restlessness, irritability, to wake with a start, and convulsion) | X  (*ira*) | X (steroid-like effect) | X (phytoecdy-steroids) |  | Frequent clinical events of toxicity in children from French Polynesia |
| *Ocimum basilicum* (aerial part) | *Puta to'e to'e* (cold, shivers, headache) | X (cold, cough, fever, phlegm) | X (analgesic, antibacterial, anti-inflammatory, antiviral, broncho-dilatory) | X (1,8-cineole, eugenol, and linalool) |  | Contraindications: < 3 years old (risk of convulsions and blanks) |
| *Persicaria glabra* (stem) | Umbilical cord care | X (inflammation) | X (antibacterial, anti-inflammatory) | X (quercetin, isorhamnetin derivatives) |  | Lack of data in children |
| *Phyllanthus amarus/debilis/tenellus/urinaria/virgatus* (aerial part) | *Tui* (abscesses, furuncles, otitis) | X (antiseptic, infections, *tui*) | X (analgesic, antibacterial, anti-inflammatory, anti-oedematogenic antiviral) | X (hinokinin, niranthin, nirtetralin, phyllanthin, phyltetralin, virgatusin) |  | Lack of data in children |
| *Psidium guajava* (leaf) | Ranula  (salivary cyst) | X (inflammation) | X (analgesic, anti-inflammatory, antipyretic) | X (catechin, gallic acid, lycopene) | X (knee pain, menstrual pain) | Lack of data in children |
| *Rorippa sarmentosa* (leaf) | Ranula  (salivary cyst) | X (inflammation) | X (anti-inflammatory, anti-edema) | X  (glucosinolates) |  | Lack of data in children  Used as food |
| *Rosa* sp. (flower) | Sinusitis |  |  |  |  | Lack of data in children |
| *Spondias dulcis* (immature fruit) | Ranula  (salivary cyst) | X (inflammation) |  |  |  | Lack of data in children |
| *Spondias dulcis* (immature fruit) | Cough |  |  |  |  | Lack of data in children  Used as food (mature fruits) |
| *Syzygium malaccense* (leaf) | *He'a* (detoxifying agent, skin disorders and disorders of the genitourinary system) | X (*he'a*, internal illnesses, urinary tract infections) | X (anti-inflammatory, antioxidant, estrogenic, thrombolytic ) | X (anacardic and ginkgolic acids, myricitrin, quercetin) |  | Precautions: sugar overload in the *he'a* remedy Lack of data in children |
| *Thespesia populnea* (fruit) | Fever | X (inflammation, infections) | X (analgesic, anti-inflammatory, antipyretic) | X (rutin, quercetin, luteolin, hesperidine) |  | Risk of contact dermatitis. Presence of cytotoxic compounds.  Lack of data in children |
