# Supplementary material for: Traditional medical practices for children in five islands from the Society archipelago (French Polynesia)
Source: J Ethnobiol Ethnomed. 2023 Oct 18;19:44. doi: 10.1186/s13002-023-00617-0 (PMC10585756; doi:10.1186/s13002-023-00617-0)
Supplement: Supplementary file 4 — Additional file 4: Table S4. Overview of the number of ingredients per remedy for the most cited children illnesses. [file 13002_2023_617_MOESM4_ESM.docx]

**Traditional medical practices for childhood diseases in five islands from the Society archipelago (French Polynesia)**

CHASSAGNE François^1,2*^, BUTAUD Jean-François^3^, HO Raimana^4^, CONTE Eric^2^, HNAWIA Édouard^5^, RAHARIVELOMANANA Phila^4^

^1^ UMR 152 PharmaDev, Université Paul Sabatier, Institut de Recherche pour le Développement (IRD), Toulouse, France

^2^ Maison des Sciences de l’Homme du Pacifique (UAR 2503), Université de la Polynésie Française / Centre National de la Recherche Scientifique, Tahiti, Polynésie Française

^3^ Correspondant du Muséum National d’Histoire Naturelle (PatriNat), Paris & Consultant en foresterie et botanique polynésienne, Tahiti, Polynésie française

^4^ UMR 214 EIO, Université de Polynésie Française, IFREMER, ILM, IRD, BP 6570, F-98702 Faaa, Tahiti, Polynésie française.

^5^ UMR 152 PharmaDev, Institut de Recherche pour le Développement (IRD), Noumea, New Caledonia.

*Corresponding author :

François Chassagne

Université Paul Sabatier

Faculté de Pharmacie

35 Chemin des Maraîchers

31062 Cedex 09

Toulouse

FRANCE

[francois.chassagne@ird.fr](mailto:francois.chassagne@ird.fr)

**Additional file 4: Table S4:** Overview of the number of ingredients per remedy for the most cited children illnesses

| **Diseases** | **Number of remedies** | **Number of remedies with one ingredient** | **Number of remedies with two ingredients** | **Number of remedies with three ingredients** | **Number of remedies with four ingredients** | **Number of remedies with > 5 ingredients** |
| --- | --- | --- | --- | --- | --- | --- |
| Restlessness, irritability, jerk (ira*) | 50 | 22 | 10 | 7 | 5 | 6 |
| Sinusitis (nanu*) | 30 | 10 | 12 | 2 | 4 | 2 |
| Ranula (salivary cyst) (arero ma'a*/double tongue) | 17 | 8 | 8 | 1 | 0 | 0 |
| Teething (niho*) | 10 | 3 | 6 | 1 | 0 | 0 |
| Cough (hota*) | 15 | 5 | 5 | 3 | 1 | 1 |
| Fracture (fati*) | 14 | 5 | 5 | 1 | 1 | 2 |
| Fever (fiva*) | 14 | 8 | 5 | 0 | 1 | 0 |
| Detoxifying agent (he'a, pu'a roto*) | 13 | 0 | 2 | 3 | 3 | 5 |
| Vaginal or urethral discharge (he'a*) | 7 | 1 | 2 | 1 | 2 | 1 |
| Asthma (ahopau*) | 11 | 2 | 5 | 1 | 2 | 1 |
| Furuncles, abscess and other disorders with pus exudation (tui*) | 11 | 5 | 1 | 0 | 1 | 4 |
| Otitis (tari'a ma'i, tui*) | 13 | 4 | 7 | 1 | 1 | 0 |
| Lower abdominal disorders (tia*) | 13 | 3 | 6 | 2 | 2 | 0 |
| Skin disorders (associated with he'a) | 11 | 3 | 1 | 2 | 3 | 2 |
| Umbilical cord care (pito*) | 7 | 2 | 1 | 2 | 2 | 0 |
| Chickenpox ('ōniho*) | 5 | 5 | 0 | 0 | 0 | 0 |
| Effects of reduced temperature (puta to'eto'e*) | 7 | 5 | 0 | 2 | 0 | 0 |

Legend: * = Tahitian name
